# Global etiology of bacterial meningitis: A systematic review and meta-analysis

**DOI:** 10.1371/journal.pone.0198772

**Published:** 2018-06-11

**Authors:** Anouk M. Oordt-Speets, Renee Bolijn, Rosa C. van Hoorn, Amit Bhavsar, Moe H. Kyaw

**Affiliations:** 1 Pallas Health Research and Consultancy BV, Rotterdam, The Netherlands; 2 Sanofi Pasteur, Powai, Mumbai, India; 3 Sanofi Pasteur Inc, Swiftwater, Pennsylvania, United States of America; Public Health England, UNITED KINGDOM

## Abstract

Bacterial meningitis is a global public health concern, with several responsible etiologic agents that vary by age group and geographical area. The aim of this systematic review and meta-analysis was to assess the etiology of bacterial meningitis in different age groups across global regions. PubMed and EMBASE were systematically searched for English language studies on bacterial meningitis, limited to articles published in the last five years. The methodological quality of the studies was assessed using a customized scoring system. Meta-analyses were conducted to determine the frequency (percentages) of seven bacterial types known to cause meningitis: *Escherichia coli*, *Haemophilus influenzae*, *Neisseria meningitidis*, *Streptococcus pneumoniae*, group B *Streptococcus agalactiae*, *Staphylococcus aureus*, and *Listeria monocytogenes*, with results being stratified by six geographical regions as determined by the World Health Organization, and seven age groups.

Of the 3227 studies retrieved, 56 were eligible for the final analysis. In all age groups, *S*. *pneumoniae* and *N*. *meningitidis* were the predominant pathogens in all regions, accounting for 25.1–41.2% and 9.1–36.2% of bacterial meningitis cases, respectively. *S*. *pneumoniae* infection was the most common cause of bacterial meningitis in the ‘all children’ group, ranging from 22.5% (Europe) to 41.1% (Africa), and in all adults ranging from 9.6% (Western Pacific) to 75.2% (Africa). *E*. *coli* and *S*. *pneumoniae* were the most common pathogens that caused bacterial meningitis in neonates in Africa (17.7% and 20.4%, respectively). *N*. *meningitidis* was the most common in children aged ±1–5 years in Europe (47.0%). Due to paucity of data, meta-analyses could not be performed in all age groups for all regions.

A clear difference in the weighted frequency of bacterial meningitis cases caused by the different etiological agents was observed between age groups and between geographic regions. These findings may facilitate bacterial meningitis prevention and treatment strategies.

## 1 Introduction

Bacterial meningitis, an infectious disease characterized by infection and inflammation of the meninges, results in significant morbidity and mortality globally. [1] Bacterial meningitis can be fatal in 50% of cases if untreated. Even when diagnosed early and treated adequately, 8–15% of the patients die, typically within 24 and 48 hours of symptom onset. [1] Furthermore, 10–20% of the survivors are prone to permanent sequelae including brain damage, hearing loss, and learning disabilities. [1] In the USA, bacterial meningitis was responsible for an estimated 4100 cases and 500 deaths annually between 2003 and 2007, [2] while developing countries face the highest burden of disease. [3] The African Meningitis Belt, comprising 26 countries in the sub-Saharan region, has the highest meningitis disease rate; in 2009, an estimated 80,000 suspected cases of meningitis, resulting in more than 4000 deaths, were reported. [1, 4]

The etiologic agents responsible for bacterial meningitis vary by age group. Among neonates, most cases of bacterial meningitis are due to group B *Streptococcus agalactiae*, *Escherichia coli*, and *Listeria monocytogenes*, while most cases in children and adults are caused by *Streptococcus pneumoniae* and *Neisseria meningitidis*. [3, 5] Although *Haemophilus influenzae* is implicated in bacterial meningitis in all age groups, it is preponderant in children <5 years of age. [6, 7] Given the variability in bacterial meningitis incidence and causative agents across regions, clear differentiation between them is essential to manage cases of bacterial meningitis. [3, 5]

Bacterial meningitis can be reduced by the use of prevention strategies against these etiological agents, such as vaccination against *H*. *influenzae* type B (Hib), *S*. *pneumoniae* and *N*. *meningitidis*. Hib and pneumococcal conjugate vaccines were introduced in the 1990s and 2000s, and the implementation of additional prevention programs utilizing these vaccines has reduced the occurrence of bacterial meningitis. [8] It has been reported that the widespread use of Hib (against *H*. *influenzae*) and PCV7 (against *S*. *pneumoniae*) vaccines have together significantly reduced cases of bacterial meningitis worldwide, in both vaccinated and non-vaccinated populations. [6, 9, 10] Reductions were also seen on the introduction of vaccines against *N*. *meningitidis*, with a decline in rates in children from 13.5 per 100,000 in 1968–1985 to 5.2 per 100,000 in 1989–2011. [11, 12] Currently there are no vaccines available against *E*. *coli*, *L*. *monocytogenes*, or group B *S*. *agalactiae*. [13, 14] Other prevention strategies include using chemoprophylaxis to prevent secondary disease in high-risk individuals. [13, 15]

The differences in etiology by age and region are yet to be systematically reviewed on a global scale. This systematic review and meta-analysis summarizes available data on the etiology of bacterial meningitis published in the last five years, with the aim of improving current knowledge of bacterial meningitis in different age groups and geographical regions. This could contribute to the management of the disease through development of effective prevention strategies and treatment guidelines.

## 2 Material and methods

This systematic review was conducted following the Cochrane Collaboration and Preferred Reporting Items for Systematic Reviews and Meta-Analyses (PRISMA) guidelines. [16, 17] The PRISMA checklist is included in S1 Supporting Information. This study is registered with PROSPERO (CRD42017074949).

### 2.1 Search strategy for identification of studies

A systematic search of the PubMed and EMBASE databases was conducted (search terms listed in S1 Table), and was limited to articles published in the last five years (i.e. 25 April 2012 to 25 April 2017).

### 2.2 Inclusion and exclusion criteria

To be included, studies had to report original data on the etiology of bacterial meningitis in English. Studies were excluded if they were another article type (i.e. expert opinions, letters to the editor, editorials, comments, narrative reviews, and case reports), were on a genetic/molecular level, phase I/II trials, diagnostic accuracy studies, or outbreaks. Any studies that only reported data on a specific etiological agent of bacterial meningitis, considered specific types of patients (e.g., HIV-positive or immunocompromised patients), reported data on recurrent bacterial meningitis, did not permit extraction of quantitative data (e.g., if data were presented only in the form of a figure), or when the definition of bacterial meningitis included tuberculous meningitis, were also excluded. If multiple publications reporting findings of the same study were retrieved, only the most recent or most complete publication for each data set for a specific outcome was selected. Reference lists of meta-analyses and systematic reviews were scanned to identify any missing articles that might be relevant. To reduce the risk of bias, studies with a small sample size (<50 subjects) were excluded to be more representative of the population; these were only included if data from large studies were not available for certain countries.

### 2.3 Data selection

Full-text articles were identified following a preliminary screening of titles and abstracts and were reviewed in detail. As a quality check, two independent researchers individually screened the same first 30% of titles and abstracts and assessed 10% of full-text articles, with a third researcher resolving any disagreements if required. During screening there was less than 5% discrepancy between the two researchers. For each selected article, two researchers extracted the following data to an Excel spreadsheet: study characteristics (country, design, study period, and setting), study population (case definition, exclusion criteria, sample size, age, and sex), and etiological agents.

### 2.4 Risk of bias assessment

A customized checklist based on the Critical Appraisal Skills Program (CASP) [18] and on criteria relevant to the designs of studies included in the systematic review was used to assess study quality/risk of bias. The checklist included eight questions that could be answered ‘yes’, ‘no’, or ‘cannot tell/not applicable’ and were given a weight of 10 or 15 points based on relevance (S2 Table). Each study was given an overall quality assessment score based on answers to the eight questions; 100 points were scored if all eight responses were positive. Overall study quality was categorized as ‘high’, ‘moderate’, or ‘low’ when studies received scores of ≥80 points, >50 to <80 points, or ≤50 points, respectively.

Data were stratified by six geographic regions in accordance with the World Health Organization (WHO) regional classification of Member States (African region, Region of the Americas, South-East Asian region, European region, Eastern Mediterranean region [including Israel], and Western Pacific region). [19] The data were also stratified by age categories: all ages, all children (<18 years), all adults, neonates (aged <1 month), children aged ±1 month–1 year, children aged ±1–5 years, children aged ±6–18 years, adults aged ±18–29 years, adults aged ±30–49 years, adults aged ±50–64 years, and elderly (±65 years). There was heterogeneity between the studies with regard to the age ranges used and therefore the authors were not too strict on the limits used in order to compare studies in children and adults.

### 2.5 Statistical analyses

Meta-analyses were performed for the frequency (percentages) of seven pathogens known to cause bacterial meningitis (*E*. *coli*, *H*. *influenzae*, *N*. *meningitidis*, *S*. *pneumoniae*, group B *S*. *agalactiae*, *S*. *aureus*, and *L*. *monocytogenes*). Analyses were performed if data stratified by pathogen, age, and region were available from at least three studies. Analyses were also performed on the frequency of the pathogens in the Northern American region (i.e. the USA and Canada), stratified by age and region to determine the impact that vaccine coverage may have. The frequency of the seven pathogens in the ‘all ages’ group was also analyzed, stratified by quality assessment score (low, moderate, or high). Additional analyses were carried out to stratify studies that investigated the frequency of group B *S*. *agalactiae* in neonates and in the ‘all ages’ group, ignoring the n≥3 studies criterion. The separate analyses of studies in the Northern American region and the frequency of the pathogens stratified by quality assessment score were carried out when more than two studies provided data for a pathogen.

To combine the study results, two approaches were used; the random-effects model was used for studies with moderate or high heterogeneity, and the fixed-effect model for those with low heterogeneity. [20–22] The inverse-variance method was used for the fixed-effect models to pool summary measures, [23] and the DerSimonian and Laird method for the random-effects models. [24–26] To prevent the exclusion of study estimates that were 0, the Freeman-Tukey transformation was used. [27, 28]

The level of heterogeneity was assessed by the Cochran’s Q test. Heterogeneity was quantified by the Higgins I^2^ test. [16] Heterogeneity was classed as low (I^2^ of 0–40%), moderate (30–60%), substantial (50–90%), or high (75–100%). [24, 29] P-values were obtained by comparing the statistic with the Chi^2^ distribution with k-1 degrees of freedom, with ≤0.10 considered the cut-off for statistically significant heterogeneity. [24] Statistical analyses were performed in STATA version 13.1 (College Station, Texas, USA).

### 2.6 Sensitivity analyses

Sensitivity analyses were performed to determine the origin of any differences between studies. Studies were omitted from the sensitivity analysis if they: reported results only on isolates or episodes instead of cases, were of low quality, reported on relatively small subgroups, or those with subgroups that excluded neonates in the ‘all ages’ and ‘all children’ groups. Sensitivity analyses were only performed when at least four studies per subgroup could be pooled after study exclusion.

## 3 Results and discussion

### 3.1 Study characteristics

**Fig 1:**
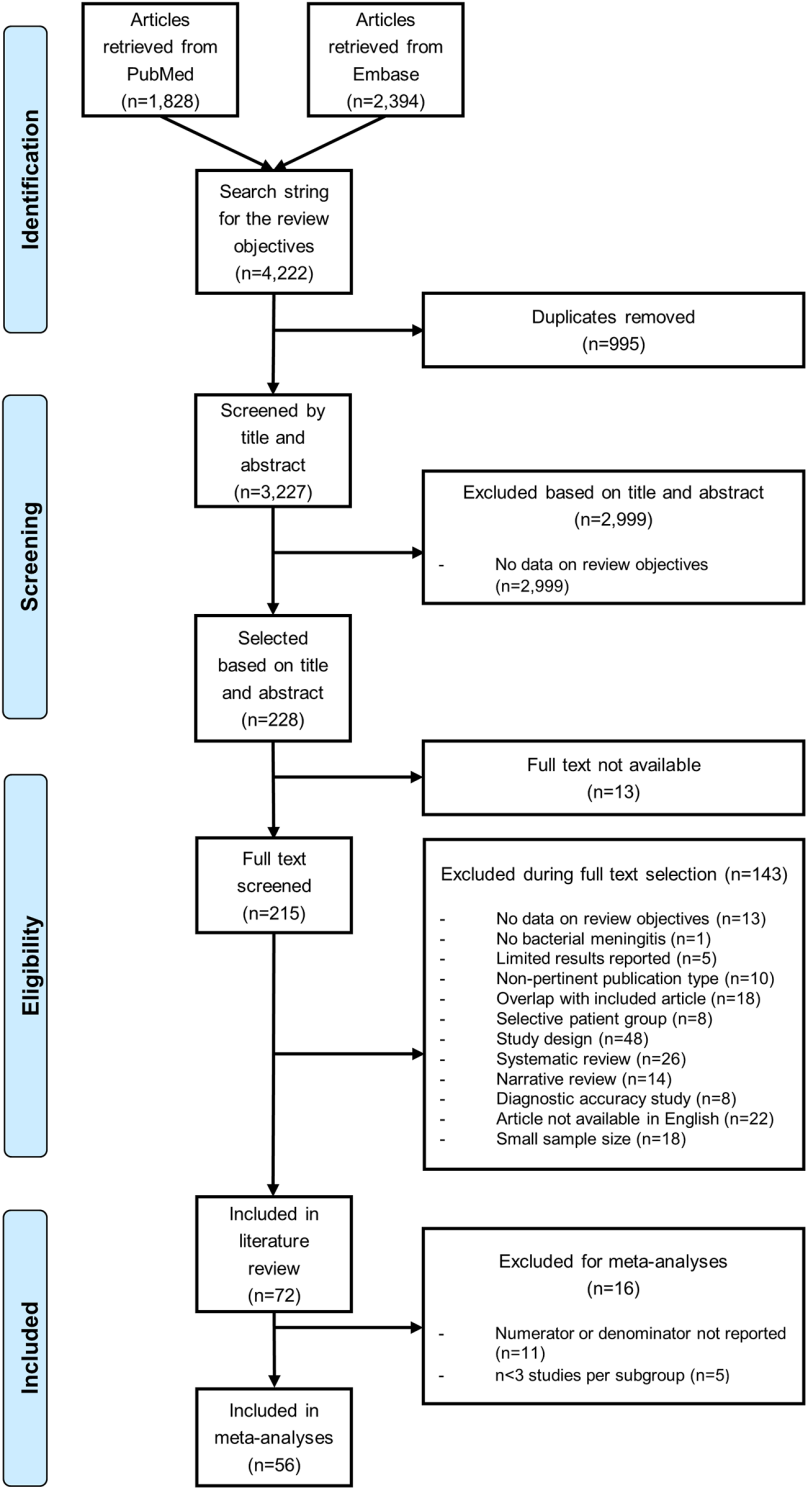
Flow chart for study inclusion.

Out of a total of 3227 unique records screened, 72 studies reported data on the etiology of bacterial meningitis by age and region (Fig 1).

Of the total 72 studies initially identified, 29 were in adults and children, 33 in only children, and 10 in only adults. Between 18 and 110,264 confirmed bacterial meningitis cases were observed in these 72 studies. Five studies of the 72 had a sample size <50 and were included because large studies were not available for those countries: Bosnia and Herzegovina, [30] Nepal, [31] Nigeria, [32] Romania, [33] and Tunisia. [34] The methodological quality assessment of each of the included studies is shown in S3 Table. Approximately 90% of these studies were cross-sectional studies (n = 49/72) or surveillance studies (n = 14/72). The majority of the studies received a ‘moderate’ quality assessment score (n = 40/72), mostly because the study populations were not representative of the source population, and overall quality assessment scores were generally comparable across geographic regions (Table 1). Ten studies received a ‘low’ quality assessment score due to insufficient case definitions and lack of adjustment for potential confounding factors, in addition to lack of representativeness of the study populations.

**Table 1:** Overall study quality assessment scores in each geographic region.

	Study quality score		
Geographic region, n (%)	Low	Moderate	High
African region	3 (16.7)	11 (61.1)	4 (22.2)
Region of the Americas	1 (16.7)	2 (33.3)	3 (50.0)
South-East Asia region	2 (40.0)	2 (40.0)	1 (20.0)
European region	1 (5.6)	11 (61.1)	6 (33.3)
Eastern Mediterranean region	2 (15.4)	6 (46.2)	5 (38.5)
Western Pacific region	1 (8.3)	8 (66.7)	3 (25.0)
**Overall**	**10 (13.9)**	**40 (55.6)**	**22 (30.6)**

### 3.2 Etiology of bacterial meningitis

Of the 72 studies included in the literature review, 61 studies were eligible for the meta-analysis (11 studies were excluded because the numerator or denominator was missing; 5 studies were excluded as these presented limited data [≤3 studies per subgroup]), resulting in the final inclusion of 56 studies.

The meta-analysis of these studies showed that the most prevalent pathogens that caused bacterial meningitis were *N*. *meningitidis*, and *S*. *pneumoniae*; with weighted means for frequency across geographical regions and age groups ranging from 3.2–47.0%, and 9.6–75.2%, respectively. The range of weighted means for frequency of *H*. *influenzae* was 0.2–15.5% (Table 2, S4 Table). *S*. *pneumoniae* was the most frequently implicated cause of bacterial meningitis in the ‘all children’ and ‘all adults’ groups, with weighted means for frequency between 22.5–41.1% and 9.6–75.2%, respectively (Table 2).

**Table 2:** Overview of studies with data on percentages of pathogens that caused bacterial meningitis stratified by age group and region*.

	*E*. *coli*	*H*. *influenzae*	*L*. *monocytogenes*	*N*. *meningitidis*	*S*. *aureus*	*Group B S*. *agalactiae*	*S*. *pneumoniae*
***All ages***							
**African region, n**	8 [35–42]	10 [35–44]	7 [35–41]	10 [35–44]	8 [35–42]	7 [35–41]	10 [35–44]
Weighted mean, %	0.99	5.59	0.00	36.18	0.89	0.23	41.17
(95% CI)	(0.00–3.18)	(3.50–8.09)	(0.00–0.11)	(26.58–46.36)	(0.00–2.85)	(0.00–1.17)	(34.10–48.43)
I^2^, % (p-value)	94.9 (p<0.001)	93.5 (p<0.001)	36.4 (p = 0.15)	98.7 (p<0.001)	94.2 (p<0.001)	88.0 (p<0.001)	97.3 (p<0.001)
**Eastern Mediterranean, n**	2	3 [45–47]	2	4 [45–48]	2	0	4 [45, 47–49]
Weighted mean, %		6.41		9.09			25.13
(95% CI)		(0.29–19.02)		(6.19–13.17)			(14.23–37.91)
I^2^, % (p-value)		NA		99.3 (p<0.001)			96.2 (p<0.001)
**Europe, n**	2	3 [50–52]	3 [50–52]	3 [50–52]	3 [50–52]	3 [50–52]	3 [50–52]
Weighted mean, %		2.36	1.63	36.18	4.36	1.93	27.0
(95% CI)		(0.88–4.43)	(0.58–3.10)	(17.91–56.78)	(0.25–12.33)	(0.00–6.14)	(7.93–52.06)
I^2^, % (p-value)		NA	NA	NA	NA	NA	NA
**South-East Asia, n**	1	2	2	2	1	1	2
**The Americas, n**	0	4 [53–56]	2	4 [53–56]	0	0	4 [53–56]
Weighted mean, %		7.84		26.94			26.23
(95% CI)		(3.84–13.09)		(22.57–31.54)			(8.21–50.12)
I^2^, % (p-value)		99.9 (p<0.001)		99.6 (p<0.001)			99.8 (p<0.001)
**Western Pacific, n**	1	2	1	2	0	0	2
***All children***							
**African region, n**	4 [32, 39, 57, 58]	4 [32, 39, 57, 58]	4 [32, 39, 57, 58]	4 [32, 39, 57, 58]	4 [32, 39, 57, 58]	4 [32, 39, 57, 58]	5 [32, 39, 42, 57, 58]
Weighted mean, %	2.45	13.14	0.00	7.46	2.00	2.93	41.06
(95% CI)	(0.00–10.14)	(0.39–37.17)	(0.00–6.80)	(1.91–15.75)	(0.00–6.50)	(0.00–10.67)	(24.66–58.52)
I^2^, % (p-value)	84.8 (p<0.001)	95.3 (p<0.001)	0.0 (p = 1.00)	77.0 (p<0.001)	67.1 (p = 0.03)	83.8 (p<0.001)	91.2 (p<0.001)
**Eastern Mediterranean, n**	1	2	1	2	1	1	1
**Europe, n**	2	8 [30, 33, 52, 59–63]	3 [30, 33, 52]	9 [30, 33, 50, 52, 59–63]	3 [30, 50, 52]	5 [30, 33, 50, 52, 59]	9 [30, 33, 50, 52, 59–63]
Weighted mean, %		13.59	0.00	46.87	2.22	2.25	22.47
(95% CI)		(6.70–22.26)	(0.00–1.35)	(35.27–58.64)	(0.03–6.57)	(0.00–7.83)	(14.13–32.05)
I^2^, % (p-value)		93.9 (p<0.001)	NA	97.3 (p<0.001)	NA	94.2 (p<0.001)	96.7 (p<0.001)
**South-East Asia, n**	2	2	2	2	2	2	2
**The Americas, n**	1	2	2	2	1	2	2
**Western Pacific, n**	5 [64–68]	5 [64–68]	2	5 [64–68]	3 [64, 66, 69]	5 [64–68]	5 [64–68]
Weighted mean, %	10.32	13.87		3.15	4.15	13.65	26.18
(95% CI)	(6.55–14.80)	(6.70–22.26)		(0.69–7.06)	(0.00–13.83)	(4.60–26.35)	(17.73–35.61)
I^2^, % (p-value)	74.0 (p<0.001)	95.5 (p<0.001)		84.9 (p<0.001)	NA	95.5 (p<0.001)	88.9 (p<0.001)
***All adults***							
**African region, n**	2	2	2	2	2	2	3 [39, 42, 70]
Weighted mean, %							75.18
(95% CI)							(56.19–90.22)
I^2^, % (p-value)							NA
**Eastern Mediterranean, n**	1	1	1	1	1	0	0
**Europe, n**	6 [50, 71–75]	5 [52, 71, 72, 74, 75]	6 [52, 71–75]	7 [50, 52, 71–75]	7 [50, 52, 71–75]	4 [52, 73–75]	7 [50, 52, 71–75]
Weighted mean, %	2.86	2.55	5.84	24.31	5.48	0.95	38.02
(95% CI)	(1.08–5.38)	(1.08–5.38)	(3.24–9.07)	(15.30–34.61)	(1.06–12.55)	(0.49–1.52)	(17.09–61.55)
I^2^, % (p-value)	93.2 (p<0.001)	0.0 (p = 0.75)	80.0 (p<0.001)	97.9 (p<0.001)	98.2 (p<0.001)	0.0 (p = 0.94)	99.6 (p<0.001)
**South-East Asia, n**	0	0	0	0	0	0	0
**The Americas, n**	0	0	0	0	0	0	0
**Western Pacific, n**	3 [68, 76, 77]	4 [68, 76–78]	4 [68, 76–78]	4 [68, 76–78]	3 [76–78]	3 [76–78]	4 [68, 76–78]
Weighted mean, %	1.82	0.20	1.10	4.59	12.16	1.28	9.55
(95% CI)	(0.35–4.15)	(0.00–1.43)	(0.00–4.03)	(0.00–22.01)	(9.00–15.70)	(0.28–2.81)	(0.92–25.01)
I^2^, % (p-value)	NA	55.3 (p = 0.08)	78.7 (p<0.001)	97.6 (p<0.001)	NA	NA	96.0 (p<0.001)

*Meta-analyses were only conducted only if there were ≥3 studies.

The frequencies of these pathogens in the other age groups; neonates, children aged ±1 month– 1 year, ±1–5 years, and ±6–18 years across geographical regions, are given in S4 Table.

n, number of studies; NA, not applicable

**Fig 2:**
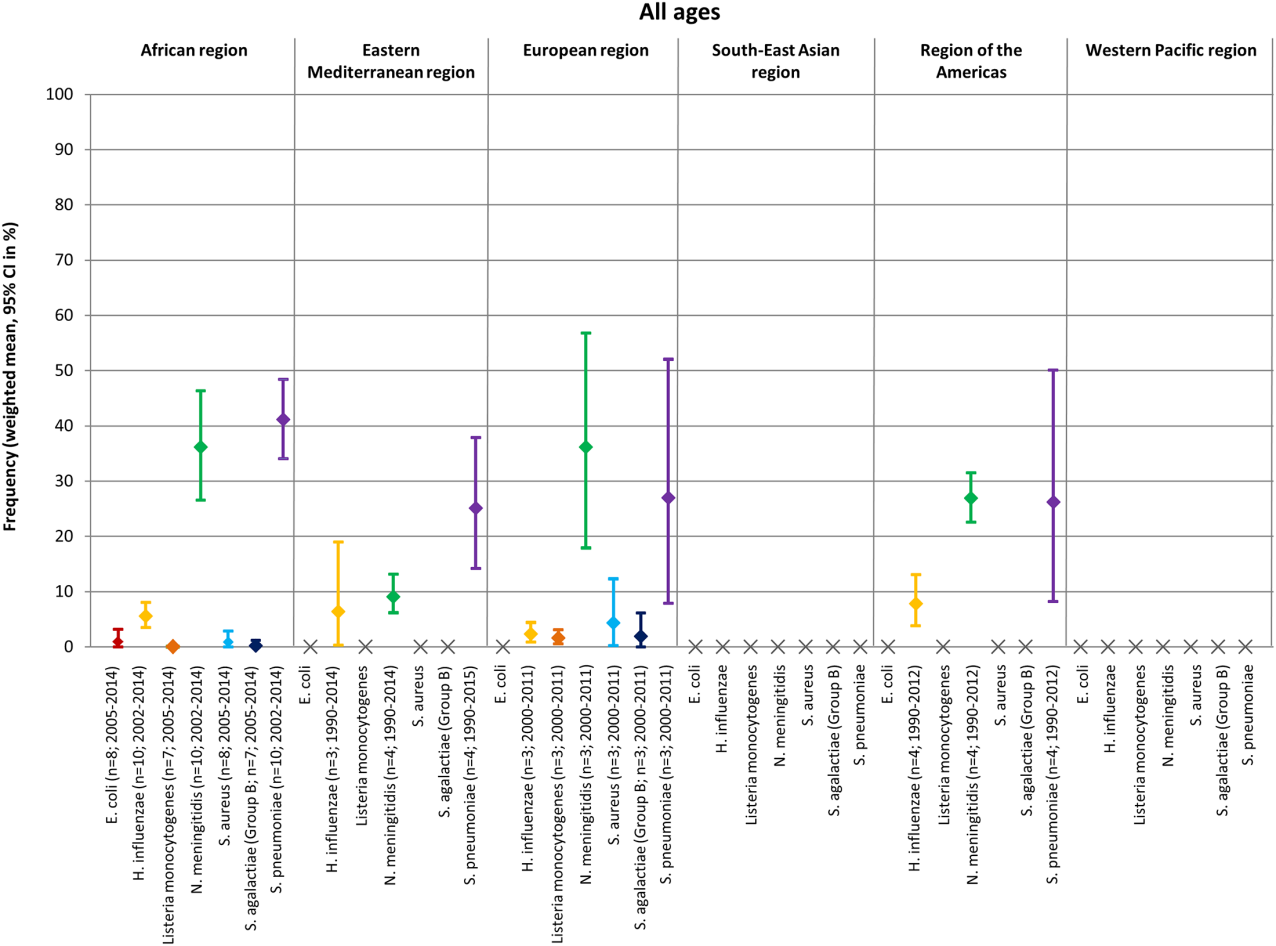
Frequency of seven pathogens that caused bacterial meningitis in all ages by geographic region.

A visual depiction of the frequency of the seven pathogens that caused bacterial meningitis stratified by age group and geographic region is provided in Fig 2 and S1 Fig.

The most common pathogens in the ‘all ages’ group, for most regions, were *N*. *meningitidis* and *S*. *pneumoniae*, with weighted means for frequency ranging from 9.1–36.2%, and from 25.1–41.2%, respectively (Fig 2); meta-analysis could not be performed for the South-east Asia or Western Pacific regions for ‘all ages’. Additional meta-analyses of two studies conducted in Northern America demonstrated that *S*. *pneumoniae* was the most common pathogen that caused bacterial meningitis in ‘all ages’, with a weighted mean of 43.1% (S5 Table; [55, 56]).

Data for meta-analyses of individual age groups by region (neonates, children aged ±1 month–1 year, aged ±1–5 years, aged ±6–18 years, adults aged ±18–29 years, aged ±30–49 years, aged ±50–64 years, and the elderly) was limited (S1 Fig). *N*. *meningitidis* was the most prevalent pathogen that caused bacterial meningitis in children aged ±1–5 years, and *S*. *pneumoniae* was the most prevalent pathogen in children aged ±6–18 years (S1 Fig). In neonates, *E*. *coli* and *S*. *pneumoniae* were the most common bacterial meningitis-causing pathogens (weighted means of 17.7% and 20.4%, respectively; S1 Fig); however, meta-analyses could only be performed in the African region. Due to the importance of investigating bacterial meningitis caused by group B *S*. *agalactiae* in neonates, additional analyses were performed for all regions and ignored the n ≥3 studies criterion for meta-analysis. The frequency of group B *S*. *agalactiae* in neonates was highest in Europe and lowest in the Eastern Mediterranean region, with weighted means of 58.2% and 4.9%, respectively (S6 Table), but frequency could not be determined in the region of the Americas.

When the prevalence of pathogens that caused bacterial meningitis in all ages was stratified by quality assessment score, *N*. *meningitidis* and *S*. *pneumoniae* remain the most prevalent pathogens in all geographic regions (data not shown).

### 3.3 Sensitivity analyses

Large heterogeneity was found between the included studies. However, most sensitivity analyses resulted in very small changes in the observed heterogeneity, indicating that these analyses did not point out specific causes for this. Clear sources of the observed heterogeneity were identified for a limited number of sensitivity analyses, although these sources did not appear to be consistent when compared with other sensitivity analyses on specific bacteria, even though the included studies were essentially the same. It is therefore unlikely that these sources of heterogeneity fully explain the observed heterogeneity between the included studies.

### 3.4 Discussion

To our knowledge, this is the first systematic review and meta-analysis of the frequency of key pathogens that cause bacterial meningitis across a wide variety of age groups and geographical regions. The present systematic review showed that *N*. *meningitidis*, *S*. *pneumoniae*, and *H*. *influenzae* were the predominant pathogens that caused bacterial meningitis in most ages within the majority of regions in studies published in the last five years.

In children aged ±6–18 years and neonates in all regions, *S*. *pneumoniae* was the predominant pathogen, and it remains an important cause of bacterial meningitis in regions with high vaccine coverage such as Northern America. [55, 56] *N*. *meningitidis* was the predominant pathogen for bacterial meningitis among children in the age group ±1–5 years in Europe. Previous studies have identified that in recent years serogroup B *N*. *meningitidis* is responsible for the majority of meningococcal disease in Europe, [79, 80] while serogroup C is the most common in the US. [80] The high frequency of bacterial meningitis cases due to *N*. *meningitidis* and *S*. *pneumoniae* come against the backdrop of the availability of vaccines for both pathogens. A decrease of ≥90% of invasive Hib disease was noted across industrialized countries following the introduction of Hib vaccination programs. [6] Administration of multiple pneumococcal conjugate vaccines resulted in a 66% reduction in the average annual rate of pneumococcal meningitis in children aged <2 years old, and a 33% reduction in adults aged ≥65 years old in the USA. [9, 10] Vaccines targeted against *N*. *meningitidis* serogroups A or C, or a tetravalent A, C, Y, and W135 vaccine have been incorporated into vaccine programs in many countries over recent years, and have led to a documented decrease in cases due to these serogroups. [11, 12] More recently a *N*. *meningitidis* serogroup B protein-based vaccine has been developed and use has been shown to limit outbreaks. [81, 82] Together these findings suggest that prevention strategies against these pathogens should be more robust, and the utilization of data on specific incidences in specific regions can help to formulate vaccination policies.

In our study, *E*.*coli* and *S*. *pneumoniae* were the most frequent pathogens that caused bacterial meningitis in neonates but this assumption was limited to meta-analyses in the African region. A retrospective analysis of bacterial meningitis in neonates and young infants between 2004 and 2014 contradicted this and found that group B *S*. *agalactiae* was the predominant pathogen, however, this was in a small population of 56 volunteers and retrospective analyses may have questionable validity. [83] An additional analysis was carried out in our study for data in neonates on the frequency of group B *S*. *agalactiae* for all regions and found that the frequency was highest in the European region (but limited to one study only [84]), and lowest in the Eastern Mediterranean region (based on two studies only [34, 85]). It has been estimated that group B *S*. *agalactiae* could cause between 114,000–204,000 invasive cases and 147,000 still births and infant deaths every year worldwide [86]. Neonatal mortality rates are estimated at 10–15% and 40–58% in developed and developing countries, respectively [87], and highlights the public health concern for group B *S*. *agalactiae*. There are no licensed vaccines against *E*. *coli* or group B *S*. *agalactiae*, despite these two pathogens being the major causes of bacterial meningitis in neonates. The availability of effective vaccines with a good tolerability profile is considered a strategic priority for the WHO to reduce the burden of invasive disease in neonates and young infants. [1, 88]

Significant heterogeneity was observed between the included studies. Sensitivity analyses omitting specific types of studies based on the types of data reported or based on study quality resulted in small changes in heterogeneity, and had little impact on the pooled estimates. It was not possible to identify the sources that fully explained the observed heterogeneity between the included studies. However, this was expected because most of the included studies were observational surveillance studies.

Our study successfully analyzed a large number of studies over a five year publication timeframe, giving a comprehensive overview of the current status of the pathogens causing bacterial meningitis worldwide. Although the search was limited to articles published in the last five years, the study periods in the included studies often covered a longer period than 2012–2017. In addition, the studies analyzed had certain limitations which might have impacted this meta-analysis. There were a small number of studies available for sub-analysis, with limited data available for some pathogens in different age groups, such as group B *S*. *agalactiae*, *L*. *monocytogenes*, *E*. *coli*, and *S*. *aureus*, highlighting the need for greater surveillance. Some studies only focused on the three most common pathogens (*H*. *influenzae*, *N*. *meningitidis*, and *S*. *pneumoniae*), while others only presented the frequency of the most common or important etiologic agent within their study population and combined the other etiologic agents in the ‘other’ category. More than half of the studies were conducted at a single institution, with a study population that may not be representative. As only studies that were published in English were included, this may have caused language bias and missed relevant studies, and may also have resulted in under-representation of the regions in our study. Some of the studies used surveillance data (n = 14/72), which is frequently incomplete and subject to systematic and random errors. Studies that provided data for specific subgroups were sometimes too small, thus meta-analyses could not be performed for all subgroups. The frequency of *N*. *meningitidis* and *S*. *pneumoniae* in all ages may be underrepresented as limited countries within the Meningitis Belt were included in this analysis. Finally, the criteria for the methodological quality assessment as well as the overall quality assessment score were customized by the authors and have not been validated.

## 4 Conclusions

This systematic review and meta-analysis demonstrated notable differences in the frequency of bacterial meningitis pathogens in a wide range of age groups across geographic regions in studies published in the last five years. Further studies are required to monitor bacterial meningitis cases and facilitate the further development of prevention and treatment strategies worldwide.

## Supporting information

S1 Supporting Information PRISMA 2009 checklist. (DOC)

S1 Fig Frequency of the seven bacteria pathogens that caused bacterial meningitis among (A) neonates, (B) children aged ±1–5 years, (C) children aged ±6–18 years by geographic region. Only analyses for frequency of pathogens in neonates, children aged ±1–5 and ±6–18 years are shown. No data were obtained in children aged ±1 month–1 year.
(PDF)

S1 Table Peer-reviewed literature search strategy. (DOCX)

S2 Table Criteria for the study quality assessment. (DOCX)

S3 Table Study characteristics for each of the included studies. CSF, cerebrospinal fluid; IQR, interquartile range; LAT, latex agglutination test; NA, not applicable; NR, not reported; PCR, polymerase chain reaction; SD, standard deviation.
(DOCX)

S4 Table Frequency of seven bacteria pathogens that caused bacterial meningitis in geographic regions, stratified by age. *The frequency of pathogens in age groups that were not presented in Table 2.
(DOCX)

S5 Table Overview of the number of studies on frequency of pathogens that caused bacterial meningitis in all ages in Northern America (USA, Canada, Greenland). n, number of studies; NA, not applicable; NR, not reported.
(DOCX)

S6 Table Overview of the number of studies on frequency of *S*. *agalactiae* group B that caused bacterial meningitis in neonates (aged <1 month), stratified by region. n, number of studies; NA, not applicable.
(DOCX)
