# Supplementary material for: Global etiology of bacterial meningitis: A systematic review and meta-analysis
Source: PLoS One. 2018 Jun 11;13(6):e0198772. doi: 10.1371/journal.pone.0198772 (PMC5995389; doi:10.1371/journal.pone.0198772)
Supplement: S1 Fig — Only analyses for frequency of pathogens in neonates, children aged ±1–5 and ±6–18 years are shown. No data were obtained in children aged ±1 month–1 year. (PDF) [file pone.0198772.s002.pdf]

Supplementary Fig 1. Frequency of the seven bacteria pathogens that caused bacterial meningitis among (A) neonates, (B) children aged ±1–5 years, (C) children aged ±6–18 years by geographic region

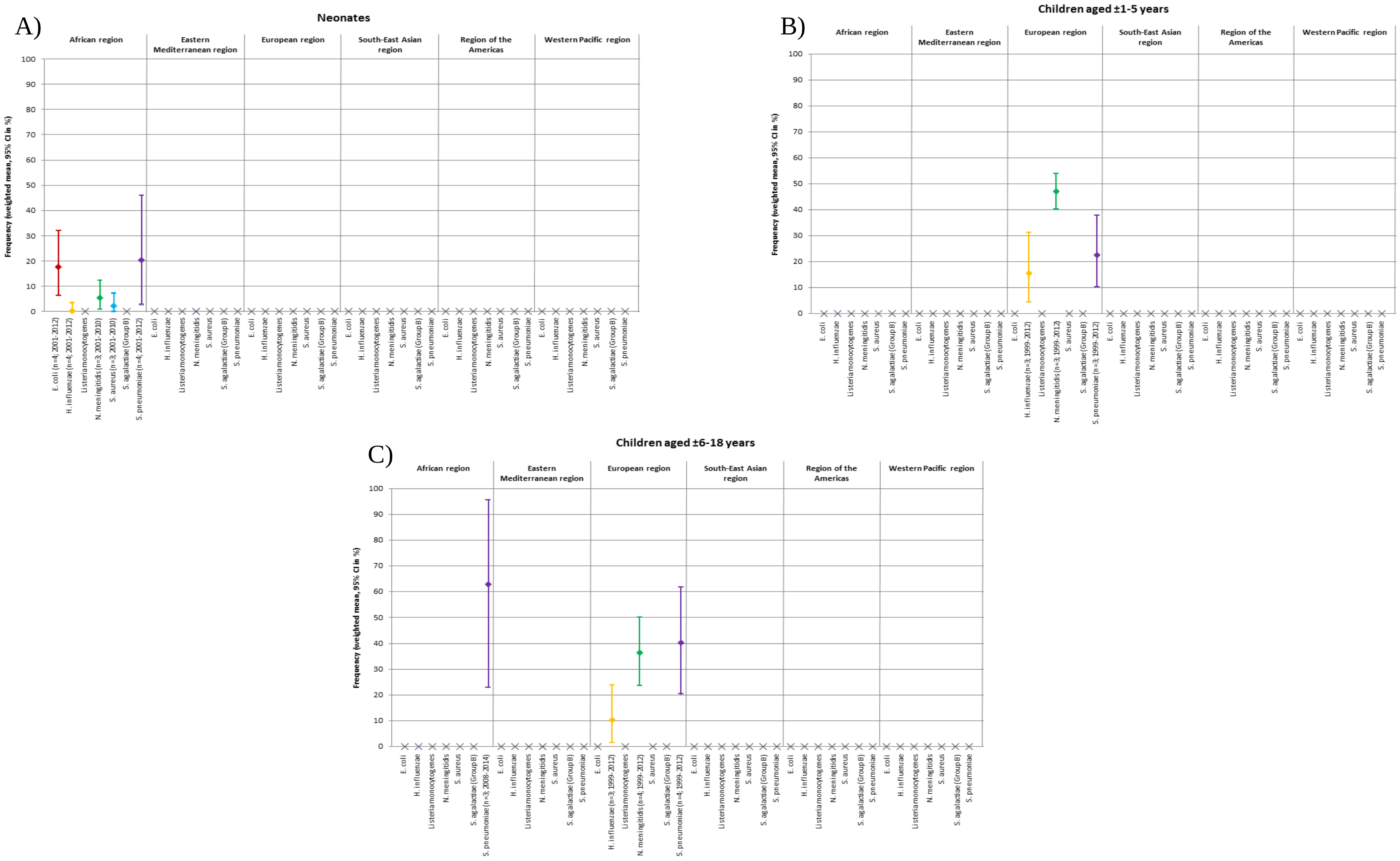

Only analyses for frequency of pathogens in neonates, children aged ±1–5 and ±6–18 years are shown. No data were obtained in children aged ±1 month–1 year.
