# Supplementary material for: Global etiology of bacterial meningitis: A systematic review and meta-analysis
Source: PLoS One. 2018 Jun 11;13(6):e0198772. doi: 10.1371/journal.pone.0198772 (PMC5995389; doi:10.1371/journal.pone.0198772)
Supplement: S1 Table — (DOCX) [file pone.0198772.s003.docx]

**S1 Table. Peer-reviewed literature search strategy.**

| **PubMed search terms** | Meningitis, Bacterial"[Mesh] OR bacterial meningitis[tiab] NOT (Animals[Mesh] NOT (Humans[Mesh] AND Animals[Mesh])) NOT (case reports[pt] OR editorial[pt] OR letter[pt] OR news[pt] OR comment[pt] OR congresses[pt]) |
| --- | --- |
| **EMBASE search terms** | 'bacterial meningitis'/exp OR 'bacterial meningitis':ti,ab NOT ([article]/lim OR [article in press]/lim OR [erratum]/lim OR [review]/lim) |
|  | Publication date April 25, 2012 to April 25, 2017 |
