# Supplementary material for: Global etiology of bacterial meningitis: A systematic review and meta-analysis
Source: PLoS One. 2018 Jun 11;13(6):e0198772. doi: 10.1371/journal.pone.0198772 (PMC5995389; doi:10.1371/journal.pone.0198772)
Supplement: S2 Table — (DOCX) [file pone.0198772.s004.docx]

**S2 Table. Criteria for the study quality assessment.**

| 1. Did the study address a clearly focused issue?  2. Was the study population selected in an acceptable way?  3. Was a proper method used for the case detection?  4. Was a proper method used for the case definition?  5. Was the outcome accurately measured to minimize bias?  6. Have the authors taken account of the potential confounding factors in the design and/or in their analysis?  7. In case of follow-up, was the follow-up of the subjects:  a. Complete enough?  b. Long enough?  8. Is the population a representative sample of the source population? |
| --- |
| Questions 2, 3, 4, and 8 were worth 15 points each when the answer was ‘yes’, whereas questions 1, 5, 6 and 7 were worth 10 points each for positive answers. No points were assigned for negative or uncertain responses. |
