# Supplementary material for: Global etiology of bacterial meningitis: A systematic review and meta-analysis
Source: PLoS One. 2018 Jun 11;13(6):e0198772. doi: 10.1371/journal.pone.0198772 (PMC5995389; doi:10.1371/journal.pone.0198772)
Supplement: S3 Table — CSF, cerebrospinal fluid; IQR, interquartile range; LAT, latex agglutination test; NA, not applicable; NR, not reported; PCR, polymerase chain reaction; SD, standard deviation. (DOCX) [file pone.0198772.s005.docx]

**S3 Table. Study characteristics for each of the included studies.**

| **Reference (first author, year); country** | **Study design; period; setting** | **Study population; sample size; sex; mean/median age** | **Case definition** | **Number of confirmed cases^1^; sex; mean/median age** | **Study quality assessment^2^** | | | | | | | | | | | | | | | | | **Overall score** | | |
| --- | --- | --- | --- | --- | --- | --- | --- | --- | --- | --- | --- | --- | --- | --- | --- | --- | --- | --- | --- | --- | --- | --- | --- | --- |
|  |  |  |  |  | **Q1** | **Q2** | | **Q3** | | **Q4** | | **Q5** | | **Q6** | | **Q7a** | | **Q7b** | | **Q8** | |  |  |  |
| **African region** | | | | | | | | | | | | | | | | | | | | | | | | |
| Kambiré, 2016 [[1](#_ENREF_1)]  Burkina Faso | Surveillance  2011-2013  Burkina Faso's nationwide bacterial meningitis surveillance system | All cases (age range: NR) registered in the bacterial meningitis surveillance system (only cases with *S. pneumoniae, N. meningitidis,* or *H. influenzae* were included in this study)  N = 12,169  Male: NR  Age: NR | Clinical diagnosis with laboratory confirmation  *Laboratory methods*  CSF analyses and culture or PCR/LAT | N = 2,858  Male: NR  Age: NR | Yes | Yes | | Yes | | Yes | | Yes | | Yes | | Can’t tell /NA | | Can’t tell /NA | | Yes | | High | | |
| Ky-Ba, 2016 [[2](#_ENREF_2)]  Burkina Faso | Surveillance  2005-2014  13 health regions in Burkina Faso (National Reference Laboratory for meningitis and 4 national laboratories) | All cerebrospinal meningitis cases (age unknown) registered in the Ministry of Health database (national epidemiological surveillance system)  N = 88,057  Male: NR  Age: NR | WHO definition  *Laboratory methods*  CSF analyses | N = 5,775  Male: NR  Age: NR | Yes | | Yes | | Yes | | Yes | | Yes | | No | | Can’t tell /NA | | Can’t tell /NA | | Yes | | High |  |
| Gervaix, 2012 [[3](#_ENREF_3)]  Cameroon | Cross-sectional  2008-2009  3 hospitals throughout Cameroon | Children (2 months - 15 years) hospitalized with fever (>38°C), clinical signs and symptoms of meningitis, and a lumbar puncture showing a turbid aspect with more than 50 polymorphonuclear cells/mm^3^ (only cases with *S. pneumoniae, N. meningitidis,* or *H. influenzae* were included in this study)  N = 170  Male: 58.2%  Median 15 (IQR 6-48) months | Clinical diagnosis with laboratory confirmation  *Laboratory methods*  CSF analyses and PCR | N = 112  Male: NR  Age: NR | Yes | | Yes | | Yes | | Yes | | Yes | | Yes | | Can’t tell /NA | | Can’t tell /NA | | Yes | | High |  |
| Massenet, 2013 [[4](#_ENREF_4)]  Cameroon | Surveillance  2007-2010  1 laboratory in northern Cameroon | Patients (age unknown) presenting with a clinical picture meeting the definition of acute meningitis  N = 1,429  Male: NR  Age: NR | Clinical diagnosis with laboratory confirmation  *Laboratory methods*  CSF culture, Gram stain, or LAT/PCR | N = 292  Male: NR  Age: NR | Yes | | Yes | | Yes | | Yes | | Yes | | No | | Can’t tell /NA | | Can’t tell /NA | | Can’t tell /NA | | Moderate |  |
| Reta, 2016 [[5](#_ENREF_5)]  Ethiopia | Cross-sectional  2001-2010  1 hospital in Addis Abeba | All neonates (≤28 days) suspected with bacterial meningitis infection  N = 1,189  Male: 53.2%  Age: NR | Laboratory diagnosis  *Laboratory methods*  CSF culture | N = 56  Male: NR  Age: NR | Yes | | Yes | | Yes | | No | | Yes | | Yes | | Can’t tell /NA | | Can’t tell /NA | | No | | Moderate |  |
| Tegene, 2015 [[6](#_ENREF_6)]  Ethiopia | Cross-sectional  2006-2010  1 hospital in northern Ethiopia | Suspected meningitis cases (age range: NR)  N = 3,085  Male: 51.5%  Mean 2,9 (SD 1,0) years | Laboratory diagnosis  *Laboratory methods*  CSF culture | N = 120  Male: 52.5%  Age: NR | Yes | | Yes | | Yes | | No | | Yes | | No | | Can’t tell /NA | | Can’t tell /NA | | No | | Low |  |
| Nuoh, 2016 [[7](#_ENREF_7)]  Ghana | Cross-sectional  2009-2013  District Health Information Management Systems (DHIMS 2) and meningitis case based forms from the Disease Control Unit of the Upper West Regional Health Directorate | Reported cases of meningitis (age range: NR)  N = 840  Male: 48.3%  Mean 20,1 (SD 18,8) years | NR  *Laboratory methods*  NR | N = 253  Male: NR  Age: NR | Yes | | Yes | | Yes | | Can’t tell /NA | | Yes | | No | | Can’t tell /NA | | Can’t tell /NA | | Can’t tell /NA | | Low |  |
| Owusu, 2012 [[8](#_ENREF_8)]  Ghana | Cross-sectional  2008-2010  1 hospital in Kumasi | All patients (age range: NR) suspected of bacterial meningitis who underwent lumbar puncture  N = 4,955  Male: NR  Age: NR | Laboratory diagnosis  *Laboratory methods*  CSF analyses and culture or Gram stain | N = 117  Male: NR  Age: NR | Yes | | Yes | | Yes | | Yes | | Yes | | Yes | | Can’t tell /NA | | Can’t tell /NA | | No | | Moderate |  |
| Swann, 2014 [[9](#_ENREF_9)]  Malawi | Cross-sectional  2002-2008  1 hospital in southern Malawi | All CSF from young infants (≤60 days) with suspected meningitis  N = 259  Male: NR  Age: NR | Laboratory diagnosis  *Laboratory methods*  CSF culture | N = 259  Male: NR  Age: NR | Yes | | Yes | | Yes | | No | | Yes | | Yes | | Can’t tell /NA | | Can’t tell /NA | | No | | Moderate |  |
| Mengistu, 2013 [[10](#_ENREF_10)]  Namibia | Cross-sectional  2009-2012  Namibia Institute of Pathology (NIP) database | CSF samples (age range: NR) from the NIP database  N = 503 (isolates)  Male: NR  Age: NR | Laboratory diagnosis  *Laboratory methods*  CSF culture | N = 503 (isolates)  Male: NR  Age: NR | Yes | | Yes | | Yes | | No | | Yes | | Yes | | Can’t tell /NA | | Can’t tell /NA | | Can’t tell /NA | | Moderate |  |
| Jusot, 2013 [[11](#_ENREF_11)]  Niger | Prospective cohort  2010-2011  4 regions of Niger: the capital city Niamey, Dosso, Tillabery, and Maradi | Suspected meningitis cases (≥5 years) in whom one of the three main causal agents, *N. meningitidis*, *S. pneumoniae* or *H. influenzae*, was identified in their CSF by PCR or latex test  N = 104  Male: NR  Age: NR | WHO definition with laboratory confirmation  *Laboratory methods*  PCR or latex test in CSF | N = 83  Male: 53.0%  Mean 13,7 (SD 10,1) years | Yes | | Yes | | Yes | | Yes | | Yes | | No | | Yes | | Yes | | Yes | | High |  |
| Kuti, 2015 [[12](#_ENREF_12)]  Nigeria | Cross-sectional  2011-2013  1 hospital in Ilesha | Children (1 month - 15 years) admitted and managed for bacterial meningitis  N = 81  Male: 58.3%  Median 8,0 (IQR 5,0-25,5) months | Laboratory diagnosis  *Laboratory methods*  CSF analyses or culture or Gram stain | N = 57  Male: NR  Age: NR | Yes | | Yes | | Yes | | Yes | | Yes | | Yes | | Can’t tell /NA | | Can’t tell /NA | | No | | Moderate |  |
| Nwadioha, 2013 [[13](#_ENREF_13)]  Nigeria | Cross-sectional  2006-2009  1 hospital in Kano | Pediatric patients (0-14 years) with the clinical suspicion of acute bacterial meningitis  N = 1,500  Male: NR  Age: NR | Laboratory diagnosis  *Laboratory methods*  CSF analyses, culture, and Gram stain | N = 50  Male: NR  Age: NR | Yes | | Yes | | Yes | | No | | Yes | | Yes | | Can’t tell /NA | | Can’t tell /NA | | No | | Moderate |  |
| Britz, 2016 [[14](#_ENREF_14)]  South Africa | Cross-sectional  2009-2012  27 National Health Laboratory Service (NHLS) laboratories in Gauteng | Any person (≥18 years) who had a lumbar puncture performed and a CSF specimen sent to an NHLS laboratory  N = 1,445  Male: 42.1%  Age: NR | Laboratory diagnosis  *Laboratory methods*  CSF culture | N = 1,445  Male: 42.1%  Age: NR | Yes | | Yes | | Yes | | No | | Yes | | Yes | | Can’t tell /NA | | Can’t tell /NA | | Yes | | Moderate |  |
| Wolzak, 2012 [[15](#_ENREF_15)]  South Africa | Cross-sectional  2007-2009  1 hospital in Cape town | All children (0-13 years) who had CSF examination indicating meningitis  N = 85 (episodes)  Male: NR  Age: NR | Laboratory diagnosis  *Laboratory methods*  CSF analyses, culture or Gram stain | N = 85 (episodes)  Male: NR  Age: NR | Yes | | Yes | | Yes | | Yes | | Yes | | Yes | | Can’t tell /NA | | Can’t tell /NA | | No | | Moderate |  |
| Storz, 2016 [[16](#_ENREF_16)]  Tanzania | Cross-sectional  2002-2004  1 hospital in northern Tanzania | All patients (age range: 7 days - 90 years) with neurological symptoms and/or signs including impairment of consciousness  N = 53  Male: NR  Age: NR | Clinical diagnosis with laboratory confirmation  *Laboratory methods*  CSF analyses, culture, Gram stain and LAT | N = 53  Male: NR  Age: NR | Yes | | Yes | | Yes | | Yes | | Yes | | No | | Can’t tell /NA | | Can’t tell /NA | | No | | Moderate |  |
| Karou, 2012 [[17](#_ENREF_17)]  Togo | Cross-sectional  2007-2010  1 hospital in Dapaong | Patients (1 day - 55 years) with suspected cerebrospinal infection  N = 533  Male: 59.1%  Age: NR | Laboratory diagnosis  *Laboratory methods*  CSF culture | N = 260  Male: NR  Age: NR | Yes | | Yes | | Yes | | No | | Yes | | No | | Can’t tell /NA | | Can’t tell /NA | | No | | Low |  |
| Tall, 2016 [[18](#_ENREF_18)]  Togo | Cross-sectional  2010-2014  5 hospitals in northern Togo | Patients (age range: NR) with signs and symptoms of meningitis  N = 511  Male: NR  Age: NR | Clinical diagnosis with laboratory confirmation  *Laboratory methods*  PCR or culture of CSF | N = 188  Male: 52.7%  Age: NR | Yes | | Yes | | Yes | | No | | Yes | | Yes | | Can’t tell /NA | | Can’t tell /NA | | Can’t tell /NA | | Moderate |  |
| **Region of the Americas** | | | | | | | | | | | | | | | | | | | | | | | | |
| Azevedo, 2013 [[19](#_ENREF_19)]  Brazil | Surveillance  2000-2010  National information system for notifiable diseases (SINAN) | All meningitis and meningococcemia cases (age unknown) confirmed by clinical and/or laboratory criteria reported to SINAN  N = NR  Male: NR  Age: NR | Clinical diagnosis with laboratory confirmation  *Laboratory methods*  CSF culture or antigen test | N = 110,264  Male: NR  Age: NR | Yes | | Yes | | Yes | | Yes | | Yes | | Yes | | Can’t tell /NA | | Can’t tell /NA | | Yes | | High |  |
| Saraiva, 2015 [[20](#_ENREF_20)]  Brazil | Surveillance  1976-2012  Surveillance System for Meningitis, involving the Coordination of Epidemiology and the Epidemiological Surveillance Unit | Meningitis cases (age range: NR) that were caused by *N. meningitidis, H. influenzae* type b (Hib), *S. pneumoniae*, and *M. tuberculosis* as well as cases of aseptic meningitis and cryptococcal meningitis in Amazonas  N = 3,009  Male: NR  Age: NR | NR  *Laboratory methods*  NR | N = 3,009  Male: NR  Age: NR | Yes | | Yes | | Yes | | Can’t tell /NA | | Yes | | No | | Can’t tell /NA | | Can’t tell /NA | | Can’t tell /NA | | Low |  |
| Elenga, 2015 [[21](#_ENREF_21)]  French Guiana | Cross-sectional  2000-2010  3 hospitals in northern French Guiana | All children (0-15 years) who had been hospitalized in one of the department’s hospitals for documented bacterial meningitis  N = 60  Male: 51.5%  Median 6 months | Clinical diagnosis with laboratory confirmation  *Laboratory methods*  CSF culture and/or antigen test | N = 60  Male: 51.5%  Median 6 months | Yes | | Yes | | Yes | | Yes | | Yes | | Yes | | Can’t tell /NA | | Can’t tell /NA | | Yes | | High |  |
| Chacon-Cruz, 2016 [[22](#_ENREF_22)]  Mexico | Cross-sectional  2010-2013  9 hospitals throughout Mexico | All patients (0-15 years) with suspected bacterial meningitis  N = 129  Male: NR  Age: NR | Laboratory diagnosis  *Laboratory methods*  CSF and/or blood culture, and CSF cytochemical analysis | N = 73  Male: NR  Age: NR | Yes | | Yes | | Yes | | No | | Yes | | Yes | | Can’t tell /NA | | Can’t tell /NA | | Yes | | Moderate |  |
| Castelblanco, 2014 [[23](#_ENREF_23)]  USA | Cross-sectional  1997-2010  Healthcare Cost and Utilization Project (HCUP) network national inpatient sample (NIS) | All principal diagnoses (age range: NR) including bacterial meningitis based on ICD 9 coding across the USA  N = 50,822  Male: NR  Age: NR | ICD-9 codes  *Laboratory methods*  NR | N = 50,822  Male: NR  Age: NR | Yes | | Yes | | Yes | | Can’t tell /NA | | Yes | | Yes | | Can’t tell /NA | | Can’t tell /NA | | Yes | | Moderate |  |
| Gounder, 2015 [[24](#_ENREF_24)]  USA, Canada, Greenland | Surveillance  2000-2010  52 laboratories (23 in Alaska, 14 in Northern Canada, 15 in Greenland) | Case-isolates (0-87.5 years) identified through population-based surveillance (only cases with *S. pneumoniae, N. meningitidis,* or *H. influenzae* were included in this study)  N = 247  Male: 53.0%  Age: NR | Clinical diagnosis with laboratory confirmation  *Laboratory methods*  Culture from CSF, blood, pleural fluid, peritoneal fluid, synovial fluid | N = 247  Male: 53.0%  Age: NR | Yes | | Yes | | Yes | | Yes | | Yes | | Yes | | Can’t tell /NA | | Can’t tell /NA | | Yes | | High |  |
| **South-East Asia region** | | | | | | | | | | | | | | | | | | | | | | | | |
| Gangane, 2013 [[25](#_ENREF_25)]  India | Cross-sectional  2010-2012  1 hospital in Gulbarga | Clinically diagnosed cases (age range: NR) of meningitis with signs of meningitis  N = 308  Male: 63.3%  Age: NR | Clinical diagnosis with laboratory confirmation  *Laboratory methods*  CSF culture and Gram stain | N = 82  Male: NR  Age: NR | Yes | | Yes | | Yes | | No | | Yes | | No | | Can’t tell /NA | | Can’t tell /NA | | No | | Low |  |
| Khan, 2014 [[26](#_ENREF_26)]  India | Cross-sectional  2009-2010  1 hospital in northern India | Pediatric patients (0-15 years) with the clinical suspicion of acute bacterial meningitis  N = 150  Male: 73.0%  Age: NR | Clinical diagnosis with laboratory confirmation  *Laboratory methods*  CSF analyses, culture, Gram stain and LAT | N = 79  Male: NR  Age: NR | Yes | | Yes | | Yes | | Yes | | Yes | | Yes | | Can’t tell /NA | | Can’t tell /NA | | No | | Moderate |  |
| Modi, 2013 [[27](#_ENREF_27)]  India | Cross-sectional  2011-2012  1 hospital in Patna | Suspected cases (age range: NR) of acute bacterial meningitis  N = 252  Male: 62.3%  Age: NR | Clinical diagnosis with laboratory confirmation  *Laboratory methods*  CSF analyses, culture, Gram stain, LAT | N = 214  Male: NR  Age: NR | Yes | | Yes | | Yes | | No | | Yes | | No | | Can’t tell /NA | | Can’t tell /NA | | No | | Low |  |
| Ramachan-dran, 2013 [[28](#_ENREF_28)]  India | Surveillance  2008-2010  4 hospitals throughout India | Children (31 days - 2 years) with clinically suspected meningitis  N = 2,912  Male: 60.9%  Age: NR | Clinical diagnosis with laboratory confirmation (WHO definition)  *Laboratory methods*  CSF analyses, culture, Gram stain and LAT | N = 89  Male: NR  Age: NR | Yes | | Yes | | Yes | | Yes | | Yes | | Yes | | Can’t tell /NA | | Can’t tell /NA | | Yes | | High |  |
| Shrestha, 2015 [[29](#_ENREF_29)]  Nepal | Cross-sectional  2012-2013  1 hospital in Kathmandu | Patients (0-15 years) with clinical features suggestive of meningitis  N = 252  Male: 55.2%  Age: NR | Clinical diagnosis with laboratory confirmation  *Laboratory methods*  CSF culture, Gram stain or LAT | N = 18  Male: 50.0%  Age: NR | Yes | | Yes | | Yes | | No | | Yes | | Yes | | Can’t tell /NA | | Can’t tell /NA | | No | | Moderate |  |
| **European region** | | | | | | | | | | | | | | | | | | | | | | | | |
| Porobic-Jahic, 2013 [[30](#_ENREF_30)]  Bosnia and Herzegovina | Cross-sectional  1999-2009  1 hospital in northern Bosnia and Herzegovina | Children (1 month - 14 years) with clinical and laboratory parameters of bacterial meningitis, and positive biochemical and microbiological findings of CSF or blood  N = 140  Male: 56.5%  Mean 3,6 (SD 3,8) years | Clinical diagnosis with laboratory confirmation  *Laboratory methods*  CSF analyses and culture | N = 49  Male: NR  Age: NR | Yes | | Yes | | Yes | | No | | Yes | | Yes | | Can’t tell /NA | | Can’t tell /NA | | No | | Moderate |  |
| Bodilsen, 2016 [[31](#_ENREF_31)]  Denmark | Cross-sectional  1998-2014  National reference laboratory of Denmark | All patients (≥17 years) with a clinical presentation strongly suggestive of community-acquired bacterial meningitis  N = 195  Male: NR  Age: NR | Clinical diagnosis with laboratory confirmation  *Laboratory methods*  CSF culture, or blood culture and CSF analyses, or Gram stain of CSF, or bacterial antigen test or 16S rRNA gene amplification | N = 173  Male: 49.0%  Median 58 (IQR 45-70) years | Yes | | Yes | | Yes | | Yes | | Yes | | Yes | | Can’t tell /NA | | Can’t tell /NA | | Yes | | High |  |
| Okike, 2014 [[32](#_ENREF_32)]  England, Wales | Surveillance  2004-2011  UK National Health Service hospital microbiology laboratories in England and Wales | Records of all positive bacterial, mycobacterial, and fungal cultures from CSF or from blood cultures in patients (age range: NR) with a clinical diagnosis of meningitis in any age  N = 6,286  Male: NR  Age: NR | Clinical diagnosis with laboratory confirmation  *Laboratory methods*  CSF or blood cultures | N = 6,286  Male: NR  Age: NR | Yes | | Yes | | No | | Yes | | Yes | | Yes | | Can’t tell /NA | | Can’t tell /NA | | Can’t tell /NA | | Moderate |  |
| Bretonnière, 2015 [[33](#_ENREF_33)]  France | Retrospective cohort  2004-2008  5 ICUs throughout France | Patients (age range: NR) admitted for community-acquired meningitis  N = 157  Male: 62.0%  Mean 45 (SD 20) years | Clinical diagnosis with laboratory confirmation  *Laboratory methods*  CSF analyses | N = 136 (n = 137 isolates)  Male: NR  Age: NR | Yes | | Yes | | Yes | | No | | Yes | | No | | Can’t tell /NA | | Yes | | Yes | | Moderate |  |
| Butsashvili, 2013 [[34](#_ENREF_34)]  Georgia | Cross-sectional  2006-2010  Sentinel surveillance for bacterial meningitis in largest children’s hospital in Tbilisi, and in infectious diseases hospital | Children <5 years of age hospitalized in largest children’s hospital in Tbilisi and adult patients (>17 years) hospitalized in infectious diseases hospital with suspected bacterial meningitis (only cases with *S. pneumoniae, N. meningitidis,* or *H. influenzae* were included in this study)  N = 100  Male: NR  Age: NR | Clinical diagnosis with laboratory confirmation  *Laboratory methods*  CSF analyses | N = 100  Male: NR  Age: NR | Yes | | Can’t tell /NA | | Yes | | Can’t tell /NA | | Yes | | No | | Can’t tell /NA | | Can’t tell /NA | | Can’t tell /NA | | Low |  |
| Lummel, 2016 [[35](#_ENREF_35)]  Germany | Cross-sectional  2007-2012  Electronic in-hospital database and clinical records | All patients (≥16 years) with acute bacterial meningitis  N = 136  Male: 40.4%  Mean 53,3 years | Clinical diagnosis with laboratory confirmation  *Laboratory methods*  CSF culture and analyses | N = 114  Male: NR  Age: NR | Yes | | Yes | | Yes | | Yes | | Yes | | Yes | | Can’t tell /NA | | Can’t tell /NA | | No | | Moderate |  |
| Snaebjarnar-dottir, 2013 [[36](#_ENREF_36)]  Iceland | Cross-sectional  1975-2010  1 hospital in Reykjavik (reference laboratory for the entire country) | Icelandic children (0-18 years) diagnosed with bacterial meningitis caused by *S. pneumoniae, H. influenzae, N. meningitidis*, or *S. agalactiae* (Group B)  N = 477  Male: 51.0%  Mean 4.9 (5.6) years; median 2.3 years | Clinical diagnosis with laboratory confirmation  *Laboratory methods*  CSF analyses, culture, antigen detection, or PCR of CSF or positive blood cultures | N = 477 (1995-2010: n=131)  Male: 51.0%  Mean 4.9 (5.6) years; median 2.3 years | Yes | | Yes | | Yes | | No | | Yes | | Yes | | Can’t tell /NA | | Can’t tell /NA | | Yes | | Moderate |  |
| Thornorethar-dottir, 2014 [[37](#_ENREF_37)]  Iceland | Cross-sectional  1995-2010  2 hospitals who receive all CSF samples sent for culture in the country | All Icelandic patients (≥16 years) with bacterial meningitis  N = 110 (n = 111 episodes)  Male: 56.8%  Mean 47 years | Clinical diagnosis with laboratory confirmation  *Laboratory methods*  CSF analyses, or culture, LAT, blood culture, Gram stain, or PCR in CSF | N = 105 (n = 106 episodes  Male: NR  Age: NR | Yes | | Yes | | Yes | | Yes | | Yes | | Yes | | Can’t tell /NA | | Can’t tell /NA | | Yes | | High |  |
| Ciofi degli Atti, 2014 [[38](#_ENREF_38)]  Italy | Cross-sectional  2009-2013  19 hospitals (mostly in northern Italy) | Children (1 month - 18 years) discharged with diagnosis of bacterial meningitis  N = 85  Male: 64.7%  Median 3,3 (IQR 0,5-7,1) years | Clinical diagnosis with laboratory confirmation  *Laboratory methods*  CSF analyses and culture or PCR from CSF or blood, or antigen detection on CSF | N = 56  Male: NR  Age: NR | Yes | | Yes | | Yes | | Yes | | Yes | | Yes | | Can’t tell /NA | | Can’t tell /NA | | No | | Moderate |  |
| Namani, 2014 [[39](#_ENREF_39)]  Kosovo | Cross-sectional  2000-2010  1 hospital in Pristina, Kosovo | Bacterial meningitis cases (1 month - 74 years)  N = 160  Male: NR  Age: NR | Clinical diagnosis with laboratory confirmation (WHO definition)  *Laboratory methods*  CSF analyses and blood or CSF culture, or LAT or CSF Gram stain | N = 82  Male: NR  Age: NR | Yes | | Yes | | Yes | | Yes | | Yes | | Yes | | Can’t tell /NA | | Can’t tell /NA | | No | | Moderate |  |
| Bijlsma, 2016 [[40](#_ENREF_40)]  The Netherlands | Cross-sectional  2006-2014  Database of the Netherlands Reference Laboratory for Bacterial Meningitis | Adults (≥17 years) who had bacterial meningitis in the Netherlands  N = 1,391 (n = 1,412 episodes)  Male: 50.0%  Median 61 (IQR 47-69) years | Laboratory diagnosis  *Laboratory methods*  CSF culture or CSF PCR/antigen test and CSF analyses | N = 1,391 (n = 1,412 episodes)  Male: 50.0%  Median 61 (IQR 47-69) years | Yes | | Yes | | Yes | | Yes | | Yes | | Yes | | Can’t tell /NA | | Can’t tell /NA | | Yes | | High |  |
| Juganariu, 2012 [[41](#_ENREF_41)]  Romania | Cross-sectional  2005-2010  1 hospital in northeastern Romania | Patients (0-18 years) diagnosed with bacterial meningitis  N = 100  Male: 58.0%  Mean 8 years | Clinical diagnosis with laboratory confirmation  *Laboratory methods*  NR | N = 21  Male: NR  Age: NR | Yes | | Yes | | Yes | | No | | Yes | | Yes | | Can’t tell /NA | | Can’t tell /NA | | No | | Moderate |  |
| Domingo, 2013 [[42](#_ENREF_42)]  Spain | Prospective cohort  1982-2010  1 hospital in Barcelona | All consecutive adults (≥15 years) with ABM diagnosed in a hospital in Barcelona  N = 623 (n = 635 isolates)  Male: 47.9%  Age: NR | Clinical diagnosis with laboratory confirmation  *Laboratory methods*  CSF culture or CSF antigen test, blood culture or Gram stain test with blood values | N = 510 (n=513 isolates)  Male: NR  Age: NR | Yes | | Yes | | Yes | | Yes | | Yes | | Yes | | Can’t tell /NA | | Can’t tell /NA | | No | | Moderate |  |
| Ceyhan, 2014 [[43](#_ENREF_43)]  Turkey | Cross-sectional  2005-2012  12 hospitals located in 7 regions of Turkey | Patients (1 month - 18 years) with suspected meningitis who were screened and hospitalized (only cases with *S. pneumoniae, N. meningitidis,* or *H. influenzae* were included in this study)  N = 1,452  Male: 64.1%  Mean 4,8 (SD 4,2) years | Clinical diagnosis with laboratory confirmation  *Laboratory methods*  CSF analyses or CSF culture, PCR, Gram stain or antigen detection test | N = 645  Male: NR  Age: NR | Yes | | Yes | | Yes | | Yes | | Yes | | Yes | | Can’t tell /NA | | Can’t tell /NA | | Yes | | High |  |
| Ceyhan, 2016 [[44](#_ENREF_44)]  Turkey | Cross-sectional  2013-2014  12 hospitals located in 7 regions of Turkey | Children (1 month - 18 years) presenting with meningitis clinical findings (only cases with *S. pneumoniae, N. meningitidis,* or *H. influenzae* were included in this study)  N = 665  Male: 56.3%  Median 3,5 (IQR 2-7) years | Clinical diagnosis with laboratory confirmation  *Laboratory methods*  CSF analyses, culture, PCR, Gram stain, or antigen detection test | N = 94  Male: NR  Age: NR | Yes | | Yes | | Yes | | Yes | | Can’t tell /NA | | Yes | | Can’t tell /NA | | Can’t tell /NA | | Yes | | High |  |
| Kavuncuoglu, 2013 [[45](#_ENREF_45)]  Turkey | Cross-sectional  2003-2010  1 hospital in Istanbul | Neonates (<30 days) with meningitis born in Kanuni Sultan Süleyman Training and Research Hospital, Istanbul, or those referred to the centre from nearby hospitals  N = 325  Male: NR  Age: NR | Clinical diagnosis with laboratory confirmation  *Laboratory methods*  CSF analyses, blood and CSF culture | N = 56  Male: NR  Age: NR | Yes | | Yes | | Yes | | No | | Yes | | Yes | | Can’t tell /NA | | Can’t tell /NA | | No | | Moderate |  |
| Toprak, 2014 [[46](#_ENREF_46)]  Turkey | Surveillance  2006-2009  37 hospitals located in 23 cities in 7 geographic regions of Turkey | Children (1 month - 16 years) admitted to an emergency room who underwent lumbar puncture for suspicion of meningitis and who met any of the CSF laboratory criteria, and PCR of CSF  N = 841  Male: 65.0%  Mean 51 (SD 47) years | Clinical diagnosis with laboratory confirmation  *Laboratory methods*  CSF analyses and PCR | N = 246  Male: NR  Age: NR | Yes | | Yes | | Yes | | Yes | | Yes | | Yes | | Can’t tell /NA | | Can’t tell /NA | | Yes | | High |  |
| Okike, 2014 [[47](#_ENREF_47)]  UK, Ireland | Surveillance  2010-2011  British Paediatric Surveillance Unit (BPSU), hospital microbiology laboratory reports, and parental reporting via meningitis support charities | All cases (<90 days) with a clinical diagnosis of bacterial meningitis  N = 364  Male: 55.0%  Median 17 (IQR 4-41) days | Clinical diagnosis with laboratory confirmation  *Laboratory methods*  NR | N = 298 (n = 302 isolates)  Male: NR  Age: NR | Yes | | Yes | | Yes | | No | | Yes | | Yes | | Can’t tell /NA | | Can’t tell /NA | | Can’t tell /NA | | Moderate |  |
| **Eastern Mediterranean region** | | | | | | | | | | | | | | | | | | | | | | | | |
| Teleb, 2013 [[48](#_ENREF_48)]  Afghanistan, Iran, Iraq, Egypt, Libya, Morocco, Pakistan, Sudan, Syria, Yemen | Surveillance  2005-2010  Bacterial meningitis surveillance (BMS) network in which 48 hospitals from 10 Eastern Mediterranean Region countries participate | All bacterial meningitis cases (>30 days) registered in the BMS network  N = ±30000  Male: NR  Age: NR | Clinical diagnosis with laboratory confirmation  *Laboratory methods*  CSF analyses, and culture or CSF-PCR or CSF antigen detection | N = 1,443  Male: NR  Age: NR | Yes | | Yes | | Yes | | Yes | | Yes | | No | | Can’t tell /NA | | Can’t tell /NA | | Yes | | High |  |
| Saeed, 2016 [[49](#_ENREF_49)]  Bahrain | Surveillance  1990-2013  National surveillance system of communicable diseases (both passive and active surveillance) | Confirmed meningitis cases (age unknown) registered in the national surveillance system  N = 264  Male: NR  Age: NR | Clinical diagnosis with laboratory confirmation  *Laboratory methods*  CSF culture | N = 264  Male: NR  Age: NR | Yes | | Yes | | Yes | | Yes | | Yes | | No | | Can’t tell /NA | | Can’t tell /NA | | Yes | | High |  |
| Abdelkader, 2017 [[50](#_ENREF_50)]  Egypt | Cross-sectional  2013-2015  3 hospitals in Cairo | Suspected meningitis cases (age range: NR)  N = 1,337  Male: NR  Age: NR | Clinical diagnosis with laboratory confirmation  *Laboratory methods*  CSF culture and Gram stain | N = 68 (n = 71 isolates)  Male: NR  Age: NR | Yes | | Yes | | Yes | | No | | Can’t tell /NA | | No | | Can’t tell /NA | | Can’t tell /NA | | Can’t tell /NA | | Low |  |
| Abdinia, 2014 [[51](#_ENREF_51)]  Iran | Cross-sectional  2003-2013  1 hospital in northwest of Iran | Suspected cases (≤13 years) of meningitis  N = 7,112  Male: NR  Age: NR | Laboratory diagnosis  *Laboratory methods*  CSF culture and Gram stain | N = 107  Male: 56.1%  Mean 4,2 years | Yes | | Yes | | Yes | | No | | Yes | | Yes | | Can’t tell /NA | | Can’t tell /NA | | Can’t tell /NA | | Moderate |  |
| Attarpour-Yazdi, 2014 [[52](#_ENREF_52)]  Iran | Cross-sectional  Period: NR  Several hospitals in Teheran | All patients (0-14 years) with clinical diagnosis of meningitis or with fever and leukocytosis or with increased levels of serum C-reactive protein  N = 182  Male: 56.0%  Age: NR | Clinical diagnosis with laboratory confirmation  *Laboratory methods*  CSF and blood cultures, Gram stain and LAT | N = 114  Male: 56.0%  Mean 61 (SD 40,2) months | Yes | | Yes | | Yes | | No | | Yes | | Yes | | Can’t tell /NA | | Can’t tell /NA | | Can’t tell /NA | | Moderate |  |
| Motamedifar, 2015 [[53](#_ENREF_53)]  Iran | Cross-sectional  2011-2013  1 hospital in South-West of Iran | Patients (1 month - 93 years) with suspected meningitis who referred to Nemazee Teaching Hospital  N = 2,229  Male: 53.7%  Mean 34 (SD 26) years | Clinical diagnosis with laboratory confirmation  *Laboratory methods*  CSF culture | N = 255  Male: NR  Age: NR | Yes | | Yes | | Yes | | Yes | | Yes | | Yes | | Can’t tell /NA | | Can’t tell /NA | | No | | Moderate |  |
| Rezaeizadeh, 2012 [[54](#_ENREF_54)]  Iran | Cross-sectional  1998-2008  1 hospital in Teheran (pediatric hospital) | Microbiology laboratory records of all inpatients (children; age range: NR) which were suspected to bacterial meningitis  N = 11,269 (isolates)  Male: NR  Age: NR | Laboratory diagnosis  *Laboratory methods*  CSF culture | N = 329 (isolates)  Male: NR  Age: NR | Yes | | Yes | | Yes | | No | | Yes | | No | | Can’t tell /NA | | Can’t tell /NA | | No | | Low |  |
| Ergaz, 2013 [[55](#_ENREF_55)]  Israel | Cross-sectional  1993-2009  2 hospitals in Jerusalem | All positive blood and CSF cultures obtained from inpatient infants (age range: NR) in the two neonatal units  N = 109 (n = 117 isolates)  Male: NR  Age: NR | CDC definition; or laboratory diagnosis  *Laboratory methods*  CSF culture | N = 109 (n =117 isolates)  Male: NR  Age: NR | Yes | | Yes | | Yes | | Yes | | Yes | | Yes | | Can’t tell /NA | | Can’t tell /NA | | Can’t tell /NA | | Moderate |  |
| Sadeq, 2017 [[56](#_ENREF_56)]  Kuwait | Cross-sectional  2010-2014  6 hospitals in Kuwait | Children (28 days - 12 years) admitted to any of the six hospitals with any of the following diagnoses: meningitis, bacterial meningitis, viral meningitis, aseptic meningitis, tuberculosis meningitis, or partially-treated meningitis  N = 57  Male: NR  Age: NR | Clinical diagnosis with laboratory confirmation  *Laboratory methods*  CSF analyses, and Gram stain or culture or antigen detection | N = 57  Male: NR  Age: NR | Yes | | Yes | | Yes | | Yes | | Yes | | Yes | | Can’t tell /NA | | Can’t tell /NA | | Yes | | High |  |
| Khowaja, 2013 [[57](#_ENREF_57)]  Pakistan | Case-control  2008-2011  13 health facilities in Karachi and Hyderabad-Sindh | Children (<5 years) suspected of meningitis (only cases with *S. pneumoniae* or *H. influenzae* were included in this study)  N = 188  Male: 52.5%  Mean 16,5 (SD 10,9) months | Clinical diagnosis with laboratory confirmation  *Laboratory methods*  CSF analyses and LAT and/or PCR | N = 77  Male: NR  Age: NR | Yes | | Yes | | Yes | | Yes | | Yes | | Yes | | Yes | | Can’t tell /NA | | Yes | | High |  |
| Rizvi, 2016 [[58](#_ENREF_58)]  Pakistan | Cross-sectional  2014  1 hospital in northeastern Pakistan | Cases (15-65 years) of CSF culture positive acute bacterial meningitis (only cases with *S. pneumoniae, N. meningitidis,* or *H. influenzae* were included in this study)  N = 292  Male: 61.0%  Mean 40,0 (SD 17,6) years | Clinical diagnosis with laboratory confirmation  *Laboratory methods*  CSF analyses and culture | N = 292  Male: 61.0%  Mean 40,0 (SD 17,6) years | Yes | | Yes | | Yes | | Yes | | Yes | | Yes | | Can’t tell /NA | | Can’t tell /NA | | No | | Moderate |  |
| Jarousha, 2014 [[59](#_ENREF_59)]  Palestine | Cross-sectional  2009  2 hospitals in Gaza Strip | Suspected cases (<13 years) of acute bacterial meningitis  N = 1,853  Male: 60.9%  Age: NR | Clinical diagnosis with laboratory confirmation  *Laboratory methods*  CSF analyses and culture | N = 73  Male: 62.0%  Age: NR | Yes | | Yes | | Yes | | Yes | | Yes | | Yes | | Can’t tell /NA | | Can’t tell /NA | | Yes | | High |  |
| Kamoun, 2015 [[60](#_ENREF_60)]  Tunisia | Prospective cohort  1990-2012  1 hospital in eastern Tunisia | Cases (<29 days) with neonatal purulent meningitis  N = 55  Male: 63.6%  Mean 9 days | Laboratory diagnosis  *Laboratory methods*  CSF analyses or culture | N = 30  Male: NR  Age: NR | Yes | | Yes | | Yes | | No | | Yes | | Yes | | Can’t tell /NA | | Can’t tell /NA | | No | | Moderate |  |
| **Western Pacific region** | | | | | | | | | | | | | | | | | | | | | | | | |
| Guo, 2016 [[61](#_ENREF_61)]  China | Cross-sectional  2010-2014  1 hospital in Beijing | All patients (29 days - 15 years) with probable or confirmed bacterial meningitis  N = 507  Male: 64.3%  Median 5 months | Clinical diagnosis with laboratory confirmation  *Laboratory methods*  CSF analyses, culture, and Gram stain | N = 220  Male: 60.4%  Median 6,9 (IQR 2,9-17,6) months | Yes | | Yes | | Yes | | Yes | | Yes | | Yes | | Can’t tell /NA | | Can’t tell /NA | | No | | Moderate |  |
| Li, 2014 [[62](#_ENREF_62)]  China | Surveillance  2006-2009  Acute Meningitis and Encephalitis Syndrome Project hospitals from 4 prefectures in Eastern China | All patients (age range: NR) with acute meningitis and encephalitis syndrome (only cases with *S. pneumoniae, N. meningitidis,* or *H. influenzae* were included in this study)  N = 833  Male: NR  Age: NR | Clinical diagnosis with laboratory confirmation  *Laboratory methods*  CSF analyses, blood or CSF culture, and PCR | N = 74  Male: NR  Age: NR | Yes | | Yes | | Yes | | Yes | | Yes | | No | | Can’t tell /NA | | Can’t tell /NA | | Can’t tell /NA | | Moderate |  |
| Shinjoh, 2014 [[63](#_ENREF_63)]  Japan | Cross-sectional  2011-2012  312 institutes in all areas of Japan | Cases (0-18 years) of pediatric and neonatal bacterial meningitis reported at each institute  N = 357  Male: 55.5%  Age: NR | Laboratory diagnosis  *Laboratory methods*  Culture or PCR in CSF, or pleocytosis in CSF with blood culture | N = 357  Male: 55.5%  Age: NR | Yes | | No | | Yes | | No | | Yes | | Yes | | Can’t tell /NA | | Can’t tell /NA | | No | | Low |  |
| Basri, 2015 [[64](#_ENREF_64)]  Malaysia | Cross-sectional  2004-2011  1 hospital in northern Malaysia | Patients (0-19 years) diagnosed to have bacterial meningitis with positive culture of the CSF specimen  N = 141  Male: NR  Age: NR | Laboratory diagnosis  *Laboratory methods*  CSF culture | N = 125  Male: 65.6%  Mean 74,5 (SD 80,6) months | Yes | | Yes | | Yes | | No | | Yes | | Yes | | Can’t tell /NA | | Can’t tell /NA | | No | | Moderate |  |
| Scott, 2013 [[65](#_ENREF_65)]  Mongolia | Surveillance  2002-2010  6 sites in Ulaanbaatar, the capital of Mongolia | Children (2 months - 5 years) hospitalized with suspected meningitis  N = 510  Male: NR  Age: NR | Clinical diagnosis with laboratory confirmation  *Laboratory methods*  CSF analyses and CSF or blood cultures or CSF by LAT or real time PCR | N = 254  Male: NR  Age: NR | Yes | | Yes | | Yes | | Yes | | Yes | | Yes | | Can’t tell /NA | | Can’t tell /NA | | Yes | | High |  |
| McBride, 2015 [[66](#_ENREF_66)]  New Zealand | Cross-sectional  2000-2009  1 hospital in Auckland | All cases of meningitis in adults (≥18 years) at Middlemore Hospital  N = NR  Male: NR  Age: NR | ICD-10 codes and laboratory confirmation  *Laboratory methods*  CSF analyses | N = 116 (episodes)  Male: NR  Age: NR | Yes | | Yes | | Yes | | No | | Yes | | Yes | | Can’t tell /NA | | Can’t tell /NA | | No | | Moderate |  |
| Wee, 2016 [[67](#_ENREF_67)]  Singapore | Retrospective cohort  1998-2013  1 hospital in Singapore | Children (0-18 years) admitted with acute bacterial meningitis  N = 109 (n = 112 episodes)  Male: 54.1%  Median 0,3 (0,08-1,9) years | Laboratory diagnosis  *Laboratory methods*  CSF culture, latex-based antigen detection or PCR | N = 109 (n = 112 episodes)  Male: 54.1%  Median 0,3 (0,08-1,9) years | Yes | | Yes | | Yes | | No | | Yes | | Yes | | Can’t tell /NA | | Can’t tell /NA | | No | | Moderate |  |
| Kim, 2012 [[68](#_ENREF_68)]  South Korea | Prospective cohort  2000-2010  1 hospital in Daegu | Patients (≥19 years) who were diagnosed with nosocomial meningitis  N = 91 (n=107 isolates)  Male: 54.9%  Mean 52,6 years | Laboratory diagnosis  *Laboratory methods*  CSF analyses or culture | N = 91 (n=107 isolates)  Male: 54.9%  Mean 52,6 years | Yes | | Yes | | Yes | | Yes | | Yes | | Yes | | Yes | | Yes | | No | | High |  |
| Lien, 2017 [[69](#_ENREF_69)]  Taiwan | Cross-sectional  2006-2015  1 hospital in southern Taiwan | Adult patients (≥19 years) with culture-proven bacterial meningitis  N = 157 (n=170 isolates)  Male: 61.1%  Age: NR | Clinical diagnosis with laboratory confirmation  *Laboratory methods*  CSF analyses or culture | N = 157 (n=170 isolates)  Male: 61.1%  Age: NR | Yes | | Yes | | Yes | | Yes | | Yes | | Yes | | Can’t tell /NA | | Can’t tell /NA | | No | | Moderate |  |
| Lin, 2015 [[70](#_ENREF_70)]  Taiwan | Retrospective cohort  1984-2012  1 hospital in northern Taiwan | Hospitalized patients (0-18 years) with a positive CSF bacterial culture results  N = 323 (episodes)  Male: 59.2%  Age: NR | Laboratory diagnosis  *Laboratory methods*  CSF culture | N = 323 (episodes)  Male: 59.2%  Age: NR | Yes | | Yes | | Yes | | No | | Yes | | Yes | | Yes | | Can’t tell /NA | | No | | Moderate |  |
| Ho Dang Trung, 2012 [[71](#_ENREF_71)]  Vietnam | Prospective cohort  2007-2010  13 hospitals in central and southern Vietnam | Patients (>1 month) who presented at the infectious diseases ward, ICU or paediatric ward of participating hospitals: at least 1 month of age; fever ≥38°C, headache, neck stiffness, altered consciousness and/or focal neurological signs; and CSF sample taken  N = 452  Male: NR  Age: NR | Clinical diagnosis with laboratory confirmation  *Laboratory methods*  CSF analyses, culture, Gram stain or real-time PCR of CSF sample or bacterial blood culture | N = 289  Male: NR  Age: NR | Yes | | Yes | | Yes | | Yes | | Yes | | Yes | | Can’t tell /NA | | Can’t tell /NA | | Yes | | High |  |
| Taylor, 2012 [[72](#_ENREF_72)]  Vietnam | Cross-sectional  2007-2008  1 hospital in Hanoi | Patients (>12 years) with clinical evidence of a central nervous system infection, based on the judgment of the admitting doctor  N = 113  Male: 75.2%  Age: NR | Clinical diagnosis with laboratory confirmation  *Laboratory methods*  CSF culture, Gram stain or PCR | N = 62  Male: 83.9%  Median 46 years | Yes | | Yes | | Yes | | No | | Yes | | Yes | | Can’t tell /NA | | Can’t tell /NA | | No | | Moderate |  |
| CSF, cerebrospinal fluid; IQR, interquartile range; LAT, latex agglutination test; NA, not applicable; NR, not reported; PCR, polymerase chain reaction; SD, standard deviation.   1. The number of cases were reported here, unless otherwise stated (e.g. number of isolates or episodes) 2. The study quality assessment consists of the following questions (answer categories were: yes, no, can’t tell/not applicable): 3. Did the study address a clearly focused issue? 4. Was the study population selected in an acceptable way? 5. Was a proper method used for the case detection? 6. Was a proper method used for the case definition? 7. Was the outcome accurately measured to minimize bias? 8. Have the authors taken account of the potential confounding factors in the design and/or in their analysis? 9. In case of follow-up, was the follow-up of the subjects:    1. Complete enough?    2. Long enough? 10. Is the population a representative sample of the source population? | | | | | | | | | | | | | | | | | | | | | | | | |

**References**

1. Kambire D, Soeters HM, Ouedraogo-Traore R, Medah I, Sangare L, Yameogo I, et al. Nationwide Trends in Bacterial Meningitis before the Introduction of 13-Valent Pneumococcal Conjugate Vaccine-Burkina Faso, 2011-2013. PloS one. 2016;11(11):e0166384. Epub 2016/11/11. doi: 10.1371/journal.pone.0166384. PubMed PMID: 27832151; PubMed Central PMCID: PMCPMC5104358.

2. Ky-Ba A, Sanou M, Tranchot JD, Christiasen PA, Ouedraogo AS, Tamboura M, et al. Dynamics of germs responsible for acute bacterial meningitis in Burkina faso in the last ten years (2005-2014). African Journal of Clinical and Experimental Microbiology. 2016;17(1):10-7. doi: 10.4314/ajcem.v17i1.2.

3. Gervaix A, Taguebue J, Bescher BN, Corbeil J, Raymond F, Alcoba G, et al. Bacterial meningitis and pneumococcal serotype distribution in children in cameroon. The Pediatric infectious disease journal. 2012;31(10):1084-7. Epub 2012/06/02. doi: 10.1097/INF.0b013e318260552d. PubMed PMID: 22653488.

4. Massenet D, Birguel J, Azowe F, Ebong C, Gake B, Lombart JP, et al. Epidemiologic pattern of meningococcal meningitis in northern Cameroon in 2007-2010: contribution of PCR-enhanced surveillance. Pathogens and global health. 2013;107(1):15-20. Epub 2013/02/26. doi: 10.1179/2047773212y.0000000070. PubMed PMID: 23432859; PubMed Central PMCID: PMCPMC4001598.

5. Reta MA, Zeleke TA. Neonatal bacterial meningitis in Tikur Anbessa Specialized Hospital, Ethiopia: a 10-year retrospective review. SpringerPlus. 2016;5(1):1971. Epub 2016/12/06. doi: 10.1186/s40064-016-3668-1. PubMed PMID: 27917346; PubMed Central PMCID: PMCPMC5108733.

6. Tegene B, Gebreselassie S, Fikrie N. Bacterial Meningitis: a five-year retrospective study among patients who had attended at University of Gondar Teaching Hospital, Northwest Ethiopia. Biomedical Research and Therapy. 2015;2(5). doi: 10.7603/s40730-015-0012-2.

7. Nuoh RD, Nyarko KM, Nortey P, Sackey SO, Lwanga NC, Ameme DK, et al. Review of meningitis surveillance data, upper West Region, Ghana 2009-2013. The Pan African medical journal. 2016;25(Suppl 1):9. Epub 2017/02/18. doi: 10.11604/pamj.supp.2016.25.1.6180. PubMed PMID: 28210377; PubMed Central PMCID: PMCPMC5292117.

8. Owusu M, Nguah SB, Boaitey YA, Badu-Boateng E, Abubakr AR, Lartey RA, et al. Aetiological agents of cerebrospinal meningitis: a retrospective study from a teaching hospital in Ghana. Annals of clinical microbiology and antimicrobials. 2012;11:28. Epub 2012/10/06. doi: 10.1186/1476-0711-11-28. PubMed PMID: 23035960; PubMed Central PMCID: PMCPMC3473245.

9. Swann O, Everett DB, Furyk JS, Harrison EM, Msukwa MT, Heyderman RS, et al. Bacterial meningitis in Malawian infants <2 months of age: etiology and susceptibility to World Health Organization first-line antibiotics. The Pediatric infectious disease journal. 2014;33(6):560-5. Epub 2014/01/01. doi: 10.1097/inf.0000000000000210. PubMed PMID: 24378940; PubMed Central PMCID: PMCPMC4025590.

10. Mengistu A, Gaeseb J, Uaaka G, Ndjavera C, Kambyambya K, Indongo L, et al. Antimicrobial sensitivity patterns of cerebrospinal fluid (CSF) isolates in Namibia: implications for empirical antibiotic treatment of meningitis. Journal of pharmaceutical policy and practice. 2013;6:4. Epub 2013/01/01. doi: 10.1186/2052-3211-6-4. PubMed PMID: 24764539; PubMed Central PMCID: PMCPMC3987067.

11. Jusot JF, Tohon Z, Yazi AA, Collard JM. Significant sequelae after bacterial meningitis in Niger: a cohort study. BMC infectious diseases. 2013;13:228. Epub 2013/05/22. doi: 10.1186/1471-2334-13-228. PubMed PMID: 23687976; PubMed Central PMCID: PMCPMC3664072.

12. Kuti BP, Bello EO, Jegede TO, Olubosede O. Epidemiological, clinical and prognostic profile of childhood acute bacterial meningitis in a resource poor setting. Journal of neurosciences in rural practice. 2015;6(4):549-57. Epub 2016/01/12. doi: 10.4103/0976-3147.165424. PubMed PMID: 26752902; PubMed Central PMCID: PMCPMC4692015.

13. Nwadioha SI, Nwokedi EO, Onwuezube I, Egesie JO, Kashibu E. Bacterial isolates from cerebrospinal fluid of children with suspected acute meningitis in a Nigerian tertiary hospital. The Nigerian postgraduate medical journal. 2013;20(1):9-13. Epub 2013/05/11. PubMed PMID: 23661203.

14. Britz E, Perovic O, von Mollendorf C, von Gottberg A, Iyaloo S, Quan V, et al. The Epidemiology of Meningitis among Adults in a South African Province with a High HIV Prevalence, 2009-2012. PloS one. 2016;11(9):e0163036. Epub 2016/09/27. doi: 10.1371/journal.pone.0163036. PubMed PMID: 27669564; PubMed Central PMCID: PMCPMC5036788.

15. Wolzak NK, Cooke ML, Orth H, van Toorn R. The changing profile of pediatric meningitis at a referral centre in Cape Town, South Africa. Journal of tropical pediatrics. 2012;58(6):491-5. Epub 2012/07/14. doi: 10.1093/tropej/fms031. PubMed PMID: 22791086.

16. Storz C, Schutz C, Tluway A, Matuja W, Schmutzhard E, Winkler AS. Clinical findings and management of patients with meningitis with an emphasis on Haemophilus influenzae meningitis in rural Tanzania. Journal of the neurological sciences. 2016;366:52-8. Epub 2016/06/12. doi: 10.1016/j.jns.2016.04.044. PubMed PMID: 27288776.

17. Karou SD, Balaka A, Bamoke M, Tchelougou D, Assih M, Anani K, et al. Epidemiology and antibiotic resistance of bacterial meningitis in Dapaong, northern Togo. Asian Pacific journal of tropical medicine. 2012;5(11):848-52. Epub 2012/11/14. doi: 10.1016/s1995-7645(12)60158-8. PubMed PMID: 23146796.

18. Tall H, Njanpop-Lafourcade BM, Mounkoro D, Tidjani L, Agbenoko K, Alassani I, et al. Identification of Streptococcus suis Meningitis through Population-Based Surveillance, Togo, 2010-2014. Emerging infectious diseases. 2016;22(7):1262-4. Epub 2016/06/18. doi: 10.3201/eid2207.151511. PubMed PMID: 27314251; PubMed Central PMCID: PMCPMC4918179.

19. Azevedo LC, Toscano CM, Bierrenbach AL. Bacterial Meningitis in Brazil: Baseline Epidemiologic Assessment of the Decade Prior to the Introduction of Pneumococcal and Meningococcal Vaccines. PloS one. 2013;8(6):e64524. Epub 2013/07/05. doi: 10.1371/journal.pone.0064524. PubMed PMID: 23823579; PubMed Central PMCID: PMCPMC3688798.

20. Saraiva M, Santos EC, Saraceni V, Rocha LL, Monte RL, Albuquerque BC, et al. Epidemiology of infectious meningitis in the State of Amazonas, Brazil. Revista da Sociedade Brasileira de Medicina Tropical. 2015;48 Suppl 1:79-86. Epub 2015/06/11. doi: 10.1590/0037-8682-0116-2014. PubMed PMID: 26061374.

21. Elenga N, Sicard S, Cuadro-Alvarez E, Long L, Njuieyon F, Martin E, et al. Pediatric bacterial meningitis in French Guiana. Medecine et maladies infectieuses. 2015;45(11-12):441-5. Epub 2015/11/27. doi: 10.1016/j.medmal.2015.10.003. PubMed PMID: 26607229.

22. Chacon-Cruz E, Martinez-Longoria CA, Llausas-Magana E, Luevanos-Velazquez A, Vazquez-Narvaez JA, Beltran S, et al. Neisseria meningitidis and Streptococcus pneumoniae as leading causes of pediatric bacterial meningitis in nine Mexican hospitals following 3 years of active surveillance. Therapeutic advances in vaccines. 2016;4(1-2):15-9. Epub 2016/08/24. doi: 10.1177/2051013616650158. PubMed PMID: 27551428; PubMed Central PMCID: PMCPMC4976720.

23. Castelblanco RL, Lee M, Hasbun R. Epidemiology of bacterial meningitis in the USA from 1997 to 2010: a population-based observational study. The Lancet Infectious diseases. 2014;14(9):813-9. Epub 2014/08/12. doi: 10.1016/s1473-3099(14)70805-9. PubMed PMID: 25104307.

24. Gounder PP, Zulz T, Desai S, Stenz F, Rudolph K, Tsang R, et al. Epidemiology of bacterial meningitis in the North American Arctic, 2000-2010. The Journal of infection. 2015;71(2):179-87. Epub 2015/04/14. doi: 10.1016/j.jinf.2015.04.001. PubMed PMID: 25864638; PubMed Central PMCID: PMCPMC4560175.

25. Gangane R, Praveen Kumar D. Bacteriological profile of bacterial meningitis at tertiary care hospital in north karnataka. International Journal of Pharma and Bio Sciences. 2013;4(3):B1356-B61.

26. Khan N, Malik A, Rizvi M, Afzal K, Pasha Z. Epidemiology and drug resistance profile of acute bacterial meningitis in children in Northern India: A university hospital perspective. Asian Pacific Journal of Tropical Disease. 2014;4(S2):S818-S23. doi: 10.1016/S2222-1808(14)60734-9.

27. Modi S, Anand AK. Phenotypic Characterization and Antibiogram of CSF Isolates in Acute Bacterial Meningitis. Journal of clinical and diagnostic research : JCDR. 2013;7(12):2704-8. Epub 2014/02/20. doi: 10.7860/jcdr/2013/6081.3737. PubMed PMID: 24551618; PubMed Central PMCID: PMCPMC3919387.

28. Ramachandran P, Fitzwater SP, Aneja S, Verghese VP, Kumar V, Nedunchelian K, et al. Prospective multi-centre sentinel surveillance for Haemophilus influenzae type b & other bacterial meningitis in Indian children. The Indian journal of medical research. 2013;137(4):712-20. Epub 2013/05/25. PubMed PMID: 23703338; PubMed Central PMCID: PMCPMC3724251.

29. Shrestha RG, Tandukar S, Ansari S, Subedi A, Shrestha A, Poudel R, et al. Bacterial meningitis in children under 15 years of age in Nepal. BMC pediatrics. 2015;15:94. Epub 2015/08/20. doi: 10.1186/s12887-015-0416-6. PubMed PMID: 26286573; PubMed Central PMCID: PMCPMC4541735.

30. Porobic-Jahic H, Piljic D, Jahic R, Ahmetagic S, Numanovic F. Etiology of bacterial meningitis in children in Tuzla Canton. Medical archives (Sarajevo, Bosnia and Herzegovina). 2013;67(1):13-6. Epub 2013/05/18. PubMed PMID: 23678830.

31. Bodilsen J, Dalager-Pedersen M, Schonheyder HC, Nielsen H. Time to antibiotic therapy and outcome in bacterial meningitis: a Danish population-based cohort study. BMC infectious diseases. 2016;16:392. Epub 2016/08/11. doi: 10.1186/s12879-016-1711-z. PubMed PMID: 27507415; PubMed Central PMCID: PMCPMC4977612.

32. Okike IO, Ribeiro S, Ramsay ME, Heath PT, Sharland M, Ladhani SN. Trends in bacterial, mycobacterial, and fungal meningitis in England and Wales 2004-11: an observational study. The Lancet Infectious diseases. 2014;14(4):301-7. Epub 2014/02/11. doi: 10.1016/s1473-3099(13)70332-3. PubMed PMID: 24508198.

33. Bretonniere C, Jozwiak M, Girault C, Beuret P, Trouillet JL, Anguel N, et al. Rifampin use in acute community-acquired meningitis in intensive care units: the French retrospective cohort ACAM-ICU study. Critical care (London, England). 2015;19:303. Epub 2015/08/27. doi: 10.1186/s13054-015-1021-7. PubMed PMID: 26306393; PubMed Central PMCID: PMCPMC4549935.

34. Butsashvili M, Kandelaki G, Eloshvili M, Chlikadze R, Imnadze P, Avaliani N. Surveillance of bacterial meningitis in the country of Georgia, 2006-2010. Journal of community health. 2013;38(4):724-6. Epub 2013/03/12. doi: 10.1007/s10900-013-9670-4. PubMed PMID: 23475302.

35. Lummel N, Koch M, Klein M, Pfister HW, Bruckmann H, Linn J. Spectrum and Prevalence of Pathological Intracranial Magnetic Resonance Imaging Findings in Acute Bacterial Meningitis. Clinical neuroradiology. 2016;26(2):159-67. Epub 2014/09/24. doi: 10.1007/s00062-014-0339-x. PubMed PMID: 25245328.

36. Snaebjarnardottir K, Erlendsdottir H, Reynisson IK, Kristinsson K, Halldorsdottir S, Hardardottir H, et al. Bacterial meningitis in children in Iceland, 1975-2010: a nationwide epidemiological study. Scandinavian journal of infectious diseases. 2013;45(11):819-24. Epub 2013/08/24. doi: 10.3109/00365548.2013.817680. PubMed PMID: 23968225.

37. Thornorethardottir A, Erlendsdottir H, Sigurethardottir B, Harethardottir H, Reynisson IK, Gottfreethsson M, et al. Bacterial meningitis in adults in Iceland, 1995-2010. Scandinavian journal of infectious diseases. 2014;46(5):354-60. Epub 2014/02/27. doi: 10.3109/00365548.2014.880184. PubMed PMID: 24568594.

38. Ciofi degli Atti M, Esposito S, Parola L, Rava L, Gargantini G, Longhi R. In-hospital management of children with bacterial meningitis in Italy. Italian journal of pediatrics. 2014;40:87. Epub 2015/01/15. doi: 10.1186/s13052-014-0087-1. PubMed PMID: 25584885; PubMed Central PMCID: PMCPMC4247725.

39. Namani SA, Koci RA, Qehaja-Bucaj E, Ajazaj-Berisha L, Mehmeti M. The epidemiology of bacterial meningitis in Kosovo. Journal of infection in developing countries. 2014;8(7):823-30. Epub 2014/07/16. doi: 10.3855/jidc.3553. PubMed PMID: 25022291.

40. Bijlsma MW, Brouwer MC, Kasanmoentalib ES, Kloek AT, Lucas MJ, Tanck MW, et al. Community-acquired bacterial meningitis in adults in the Netherlands, 2006-14: a prospective cohort study. The Lancet Infectious diseases. 2016;16(3):339-47. Epub 2015/12/15. doi: 10.1016/s1473-3099(15)00430-2. PubMed PMID: 26652862.

41. Juganariu G, Miftode E, Teodor D, Leca D, Dorobat CM. Clinical features and course of bacterial meningitis in children. Revista medico-chirurgicala a Societatii de Medici si Naturalisti din Iasi. 2012;116(3):722-6. Epub 2013/01/01. PubMed PMID: 23272517.

42. Domingo P, Pomar V, de Benito N, Coll P. The spectrum of acute bacterial meningitis in elderly patients. BMC infectious diseases. 2013;13:108. Epub 2013/03/01. doi: 10.1186/1471-2334-13-108. PubMed PMID: 23446215; PubMed Central PMCID: PMCPMC3599144.

43. Ceyhan M, Gurler N, Ozsurekci Y, Keser M, Aycan AE, Gurbuz V, et al. Meningitis caused by Neisseria Meningitidis, Hemophilus Influenzae Type B and Streptococcus Pneumoniae during 2005-2012 in Turkey. A multicenter prospective surveillance study. Human vaccines & immunotherapeutics. 2014;10(9):2706-12. Epub 2014/12/09. doi: 10.4161/hv.29678. PubMed PMID: 25483487; PubMed Central PMCID: PMCPMC4977434.

44. Ceyhan M, Ozsurekci Y, Gurler N, Karadag Oncel E, Camcioglu Y, Salman N, et al. Bacterial agents causing meningitis during 2013-2014 in Turkey: A multi-center hospital-based prospective surveillance study. Human vaccines & immunotherapeutics. 2016;12(11):2940-5. Epub 2016/07/28. doi: 10.1080/21645515.2016.1209278. PubMed PMID: 27454468; PubMed Central PMCID: PMCPMC5137527.

45. Kavuncuoglu S, Gursoy S, Turel O, Aldemir EY, Hosaf E. Neonatal bacterial meningitis in Turkey: epidemiology, risk factors, and prognosis. Journal of infection in developing countries. 2013;7(2):73-81. Epub 2013/02/19. doi: 10.3855/jidc.2652. PubMed PMID: 23416652.

46. Toprak D, Soysal A, Torunoglu MA, Turgut M, Turkoglu S, Pimenta FC, et al. PCR-based national bacterial meningitis surveillance in Turkey: years 2006 to 2009. The Pediatric infectious disease journal. 2014;33(10):1087-9. Epub 2014/11/02. doi: 10.1097/inf.0000000000000378. PubMed PMID: 25361189.

47. Okike IO, Johnson AP, Henderson KL, Blackburn RM, Muller-Pebody B, Ladhani SN, et al. Incidence, etiology, and outcome of bacterial meningitis in infants aged <90 days in the United kingdom and Republic of Ireland: prospective, enhanced, national population-based surveillance. Clinical infectious diseases : an official publication of the Infectious Diseases Society of America. 2014;59(10):e150-7. Epub 2014/07/06. doi: 10.1093/cid/ciu514. PubMed PMID: 24997051.

48. Teleb N, Pilishvili T, Van Beneden C, Ghoneim A, Amjad K, Mostafa A, et al. Bacterial meningitis surveillance in the Eastern Mediterranean region, 2005-2010: successes and challenges of a regional network. The Journal of pediatrics. 2013;163(1 Suppl):S25-31. Epub 2013/06/21. doi: 10.1016/j.jpeds.2013.03.027. PubMed PMID: 23773590.

49. Saeed N, AlAnsari H, AlKhawaja S, Jawad JS, Nasser K, AlYousef E. Trend of bacterial meningitis in Bahrain from 1990 to 2013 and effect of introduction of new vaccines. Eastern Mediterranean health journal = La revue de sante de la Mediterranee orientale = al-Majallah al-sihhiyah li-sharq al-mutawassit. 2016;22(3):175-82. Epub 2016/06/24. PubMed PMID: 27334074.

50. Abdelkader MM, Aboshanab KM, El-Ashry MA, Aboulwafa MM. Prevalence of MDR pathogens of bacterial meningitis in Egypt and new synergistic antibiotic combinations. PloS one. 2017;12(2):e0171349. Epub 2017/02/17. doi: 10.1371/journal.pone.0171349. PubMed PMID: 28207768; PubMed Central PMCID: PMCPMC5312949.

51. Abdinia B, Ahangarzadeh Rezaee M, Abdoli Oskouie S. Etiology and antimicrobial resistance patterns of acute bacterial meningitis in children: a 10-year referral hospital-based study in northwest iran. Iranian Red Crescent medical journal. 2014;16(7):e17616. Epub 2014/09/23. doi: 10.5812/ircmj.17616. PubMed PMID: 25237583; PubMed Central PMCID: PMCPMC4166102.

52. Attarpour-Yazdi MM, Ghamarian A, Mousaviehzadeh M, Davoudi N. Identification of the serotypes of bacterial meningitis agents; implication for vaccine usage. Iranian journal of microbiology. 2014;6(4):211-8. Epub 2015/03/25. PubMed PMID: 25802702; PubMed Central PMCID: PMCPMC4367935.

53. Motamedifar M, Ebrahim-Saraie HS, Mansury D, Nikokar I, Hashemizadeh Z. Prevalence of etiological agents and antimicrobial resistance patterns of bacterial meningitis in Nemazee Hospital, Shiraz, Iran. Archives of Clinical Infectious Diseases. 2015;10(2). doi: 10.5812/archcid.22703.

54. Rezaeizadeh G, Pourakbari B, Ashtiani MH, Asgari F, Mahmoudi S, Mamishi S. Antimicrobial susceptibility of bacteria isolated from cerebrospinal fluids in an Iranian referral pediatric center, 1998-2008. Maedica. 2012;7(2):131-7. Epub 2013/02/13. PubMed PMID: 23399784; PubMed Central PMCID: PMCPMC3557420.

55. Ergaz Z, Benenson S, Cohen MJ, Braunstein R, Bar-Oz B. No change in antibiotic susceptibility patterns in the neonatal ICU over two decades. Pediatric critical care medicine : a journal of the Society of Critical Care Medicine and the World Federation of Pediatric Intensive and Critical Care Societies. 2013;14(2):164-70. Epub 2012/12/21. doi: 10.1097/PCC.0b013e31824fbc19. PubMed PMID: 23254983.

56. Sadeq H, Husain EH, Alkoot A, Atyani S, Al-Fraij A, Al-Daithan A, et al. Childhood meningitis in Kuwait in the era of post pneumococcal conjugate vaccination: A multicenter study. Journal of infection and public health. 2017. Epub 2017/02/16. doi: 10.1016/j.jiph.2016.11.009. PubMed PMID: 28196635.

57. Khowaja AR, Mohiuddin S, Cohen AL, Khalid A, Mehmood U, Naqvi F, et al. Mortality and neurodevelopmental outcomes of acute bacterial meningitis in children aged <5 years in Pakistan. The Journal of pediatrics. 2013;163(1 Suppl):S86-S91.e1. Epub 2013/06/21. doi: 10.1016/j.jpeds.2013.03.035. PubMed PMID: 23773600.

58. Rizvi SFS, Arif U, Khan MUR. Frequency of etiological agents and the clinical outcome of acute bacterial meningitis. Pakistan Journal of Medical and Health Sciences. 2016;10(4):1264-7.

59. Jarousha AM, Afifi AA. Epidemiology and Risk Factors Associated with Developing Bacterial Meningitis among Children in Gaza Strip. Iranian journal of public health. 2014;43(9):1176-83. Epub 2015/07/16. PubMed PMID: 26175971; PubMed Central PMCID: PMCPMC4500419.

60. Kamoun F, Dowlut MB, Ameur SB, Sfaihi L, Mezghani S, Chabchoub I, et al. Neonatal purulent meningitis in southern Tunisia: Epidemiology, bacteriology, risk factors and prognosis. Fetal and pediatric pathology. 2015;34(4):233-40. Epub 2015/06/18. doi: 10.3109/15513815.2015.1051252. PubMed PMID: 26083897.

61. Guo LY, Zhang ZX, Wang X, Zhang PP, Shi W, Yao KH, et al. Clinical and pathogenic analysis of 507 children with bacterial meningitis in Beijing, 2010-2014. International journal of infectious diseases : IJID : official publication of the International Society for Infectious Diseases. 2016;50:38-43. Epub 2016/07/28. doi: 10.1016/j.ijid.2016.07.010. PubMed PMID: 27452172.

62. Li Y, Yin Z, Shao Z, Li M, Liang X, Sandhu HS, et al. Population-based surveillance for bacterial meningitis in China, September 2006-December 2009. Emerging infectious diseases. 2014;20(1):61-9. Epub 2014/01/01. doi: 10.3201/eid2001.120375. PubMed PMID: 24377388; PubMed Central PMCID: PMCPMC3884703.

63. Shinjoh M, Iwata S, Yagihashi T, Sato Y, Akita H, Takahashi T, et al. Recent trends in pediatric bacterial meningitis in Japan--a country where Haemophilus influenzae type b and Streptococcus pneumoniae conjugated vaccines have just been introduced. Journal of infection and chemotherapy : official journal of the Japan Society of Chemotherapy. 2014;20(8):477-83. Epub 2014/05/27. doi: 10.1016/j.jiac.2014.04.007. PubMed PMID: 24855913.

64. Basri R, Zueter AR, Mohamed Z, Alam MK, Norsa'adah B, Hasan SA, et al. Burden of bacterial meningitis: a retrospective review on laboratory parameters and factors associated with death in meningitis, kelantan malaysia. Nagoya journal of medical science. 2015;77(1-2):59-68. Epub 2015/03/24. PubMed PMID: 25797971; PubMed Central PMCID: PMCPMC4361508.

65. Scott S, Altanseseg D, Sodbayer D, Nymadawa P, Bulgan D, Mendsaikhan J, et al. Impact of Haemophilus influenzae Type b conjugate vaccine in Mongolia: prospective population-based surveillance, 2002-2010. The Journal of pediatrics. 2013;163(1 Suppl):S8-s11. Epub 2013/06/21. doi: 10.1016/j.jpeds.2013.03.024. PubMed PMID: 23773599.

66. McBride S, Fulke J, Giles H, Hobbs M, Suh J, Sathyendran V, et al. Epidemiology and diagnostic testing for meningitis in adults as the meningococcal epidemic declined at Middlemore Hospital. The New Zealand medical journal. 2015;128(1410):17-24. Epub 2015/04/02. PubMed PMID: 25829035.

67. Wee LY, Tanugroho RR, Thoon KC, Chong CY, Choong CT, Krishnamoorthy S, et al. A 15-year retrospective analysis of prognostic factors in childhood bacterial meningitis. Acta paediatrica (Oslo, Norway : 1992). 2016;105(1):e22-9. Epub 2015/10/02. doi: 10.1111/apa.13228. PubMed PMID: 26426265.

68. Kim HI, Kim SW, Park GY, Kwon EG, Kim HH, Jeong JY, et al. The causes and treatment outcomes of 91 patients with adult nosocomial meningitis. The Korean journal of internal medicine. 2012;27(2):171-9. Epub 2012/06/19. doi: 10.3904/kjim.2012.27.2.171. PubMed PMID: 22707889; PubMed Central PMCID: PMCPMC3372801.

69. Lien CY, Huang CR, Tsai WC, Hsu CW, Tsai NW, Chang CC, et al. Epidemiologic trend of adult bacterial meningitis in southern Taiwan (2006-2015). Journal of clinical neuroscience : official journal of the Neurosurgical Society of Australasia. 2017. Epub 2017/03/28. doi: 10.1016/j.jocn.2017.03.017. PubMed PMID: 28343916.

70. Lin MC, Chiu NC, Chi H, Ho CS, Huang FY. Evolving trends of neonatal and childhood bacterial meningitis in northern Taiwan. Journal of microbiology, immunology, and infection = Wei mian yu gan ran za zhi. 2015;48(3):296-301. Epub 2013/11/05. doi: 10.1016/j.jmii.2013.08.012. PubMed PMID: 24184002.

71. Ho Dang Trung N, Le Thi Phuong T, Wolbers M, Nguyen Van Minh H, Nguyen Thanh V, Van MP, et al. Aetiologies of central nervous system infection in Viet Nam: a prospective provincial hospital-based descriptive surveillance study. PloS one. 2012;7(5):e37825. Epub 2012/06/05. doi: 10.1371/journal.pone.0037825. PubMed PMID: 22662232; PubMed Central PMCID: PMCPMC3360608.

72. Taylor WR, Nguyen K, Nguyen D, Nguyen H, Horby P, Nguyen HL, et al. The Spectrum of Central Nervous System Infections in an Adult Referral Hospital in Hanoi, Vietnam. PLoS ONE. 2012;7(8). doi: 10.1371/journal.pone.0042099.
