# Supplementary material for: Global etiology of bacterial meningitis: A systematic review and meta-analysis
Source: PLoS One. 2018 Jun 11;13(6):e0198772. doi: 10.1371/journal.pone.0198772 (PMC5995389; doi:10.1371/journal.pone.0198772)
Supplement: S4 Table — *The frequency of pathogens in age groups that were not presented in Table 2. (DOCX) [file pone.0198772.s006.docx]

**S4 Table. Frequency of seven bacteria pathogens that caused bacterial meningitis in geographic regions, stratified by age*.**

|  | ***E. coli*** | ***H. influenzae*** | ***L. monocytogenes*** | ***N. meningitidis*** | ***S. aureus*** | ***S. agalactiae (Group B)*** | ***S. pneumoniae*** |
| --- | --- | --- | --- | --- | --- | --- | --- |
| **Geographic region** |  |  |  |  |  |  |  |
| ***Neonates*** |  |  |  |  |  |  |  |
| **African region, n**  Weighted mean, %  (95% CI)  I^2^, % (p-value) | 4 [[1-4](#_ENREF_1)]  17.66  (6.42–32.15)  50.2 (p=0.11) | 4 [[1-4](#_ENREF_1)]  0.21  (0.00–3.52)  0.0 (p=0.99) | 2 | 3 [[1](#_ENREF_1), [2](#_ENREF_2), [4](#_ENREF_4)]  5.40  (0.89–12.30)  NA | 3 [[1](#_ENREF_1), [2](#_ENREF_2), [4](#_ENREF_4)]  2.19  (0.00–7.28)  NA | 2 | 4 [[1-4](#_ENREF_1)]  20.35  (2.78–46.01)  81.7 (p<0.001) |
| **Eastern Mediterranean, n** | 2 | 1 | 2 | 1 | 2 | 2 | 1 |
| **Europe, n** | 2 | 1 | 1 | 2 | 1 | 1 | 1 |
| **South-East Asia, n** | 2 | 2 | 2 | 2 | 2 | 2 | 2 |
| **The Americas, n** | 0 | 0 | 0 | 0 | 0 | 0 | 0 |
| **Western Pacific, n** | 1 | 1 | 0 | 1 | 0 | 1 | 1 |
| ***Children aged ±1 month - 1 year*** |  |  |  |  |  |  |  |
| **African region, n** | 2 | 1 | 1 | 1 | 1 | 1 | 2 |
| **Eastern Mediterranean, n** | 0 | 0 | 0 | 0 | 0 | 0 | 0 |
| **Europe, n** | 1 | 2 | 1 | 2 | 1 | 1 | 2 |
| **South-East Asia, n** | 2 | 2 | 2 | 2 | 2 | 2 | 2 |
| **The Americas, n** | 0 | 0 | 0 | 0 | 0 | 0 | 0 |
| **Western Pacific, n** | 1 | 1 | 0 | 1 | 0 | 1 | 1 |
| ***Children aged***  ***±1-5 years*** |  |  |  |  |  |  |  |
| **African region, n** | 2 | 1 | 1 | 1 | 1 | 1 | 2 |
| **Eastern Mediterranean, n** | 0 | 0 | 0 | 0 | 0 | 0 | 0 |
| **Europe, n**  Weighted mean, %  (95% CI)  I^2^, % (p-value) | 1 | 3 [[5-7](#_ENREF_5)]  15.49  (4.44–31.07)  NA | 1 | 3 [[5-7](#_ENREF_5)]  47.04  (40.28–53.84)  NA | 1 | 1 | 3 [[5-7](#_ENREF_5)]  22.52  (10.24–37.69)  NA |
| **South-East Asia, n** | 2 | 2 | 2 | 2 | 2 | 2 | 2 |
| **The Americas, n** | 0 | 0 | 0 | 0 | 0 | 0 | 0 |
| **Western Pacific, n** | 1 | 1 | 0 | 1 | 0 | 1 | 1 |
| ***Children aged***  ***±6-18 years*** |  |  |  |  |  |  |  |
| **African region, n**  Weighted mean, %  (95% CI)  I^2^, % (p-value) | 2 | 2 | 2 | 2 | 2 | 2 | 3 [[2](#_ENREF_2), [8](#_ENREF_8), [9](#_ENREF_9)]  62.91  (22.94-95.58)  NA |
| **Eastern Mediterranean, n** | 0 | 0 | 0 | 0 | 0 | 0 | 0 |
| **Europe, n**  Weighted mean, %  (95% CI)  I^2^, % (p-value) | 1 | 3 [[5](#_ENREF_5), [7](#_ENREF_7), [10](#_ENREF_10)]  10.36  (1.59–23.77)  NA | 1 | 4 [[5](#_ENREF_5), [7](#_ENREF_7), [10](#_ENREF_10), [11](#_ENREF_11)]  36.43  (23.65–50.18)  88.3 (p<0.001) | 1 | 1 | 4 [[5-7](#_ENREF_5), [10](#_ENREF_10)]  40.28  (20.42–61.82)  95.5 (p<0.001) |
| **South-East Asia, n** | 2 | 2 | 2 | 2 | 2 | 2 | 2 |
| **The Americas, n** | 0 | 0 | 0 | 0 | 0 | 0 | 0 |
| **Western Pacific, n** | 1 | 1 | 0 | 1 | 0 | 1 | 1 |

*The frequency of pathogens in age groups that were not presented in Table 2.

**References**

1. Reta MA, Zeleke TA. Neonatal bacterial meningitis in Tikur Anbessa Specialized Hospital, Ethiopia: a 10-year retrospective review. SpringerPlus. 2016;5(1):1971. Epub 2016/12/06. doi: 10.1186/s40064-016-3668-1. PubMed PMID: 27917346; PubMed Central PMCID: PMCPMC5108733.

2. Owusu M, Nguah SB, Boaitey YA, Badu-Boateng E, Abubakr AR, Lartey RA, et al. Aetiological agents of cerebrospinal meningitis: a retrospective study from a teaching hospital in Ghana. Annals of clinical microbiology and antimicrobials. 2012;11:28. Epub 2012/10/06. doi: 10.1186/1476-0711-11-28. PubMed PMID: 23035960; PubMed Central PMCID: PMCPMC3473245.

3. Mengistu A, Gaeseb J, Uaaka G, Ndjavera C, Kambyambya K, Indongo L, et al. Antimicrobial sensitivity patterns of cerebrospinal fluid (CSF) isolates in Namibia: implications for empirical antibiotic treatment of meningitis. Journal of pharmaceutical policy and practice. 2013;6:4. Epub 2013/01/01. doi: 10.1186/2052-3211-6-4. PubMed PMID: 24764539; PubMed Central PMCID: PMCPMC3987067.

4. Nwadioha SI, Nwokedi EO, Onwuezube I, Egesie JO, Kashibu E. Bacterial isolates from cerebrospinal fluid of children with suspected acute meningitis in a Nigerian tertiary hospital. The Nigerian postgraduate medical journal. 2013;20(1):9-13. Epub 2013/05/11. PubMed PMID: 23661203.

5. Porobic-Jahic H, Piljic D, Jahic R, Ahmetagic S, Numanovic F. Etiology of bacterial meningitis in children in Tuzla Canton. Medical archives (Sarajevo, Bosnia and Herzegovina). 2013;67(1):13-6. Epub 2013/05/18. PubMed PMID: 23678830.

6. Okike IO, Ribeiro S, Ramsay ME, Heath PT, Sharland M, Ladhani SN. Trends in bacterial, mycobacterial, and fungal meningitis in England and Wales 2004-11: an observational study. The Lancet Infectious diseases. 2014;14(4):301-7. Epub 2014/02/11. doi: 10.1016/s1473-3099(13)70332-3. PubMed PMID: 24508198.

7. Ceyhan M, Gurler N, Ozsurekci Y, Keser M, Aycan AE, Gurbuz V, et al. Meningitis caused by Neisseria Meningitidis, Hemophilus Influenzae Type B and Streptococcus Pneumoniae during 2005-2012 in Turkey. A multicenter prospective surveillance study. Human vaccines & immunotherapeutics. 2014;10(9):2706-12. Epub 2014/12/09. doi: 10.4161/hv.29678. PubMed PMID: 25483487; PubMed Central PMCID: PMCPMC4977434.

8. Kuti BP, Bello EO, Jegede TO, Olubosede O. Epidemiological, clinical and prognostic profile of childhood acute bacterial meningitis in a resource poor setting. Journal of neurosciences in rural practice. 2015;6(4):549-57. Epub 2016/01/12. doi: 10.4103/0976-3147.165424. PubMed PMID: 26752902; PubMed Central PMCID: PMCPMC4692015.

9. Tall H, Njanpop-Lafourcade BM, Mounkoro D, Tidjani L, Agbenoko K, Alassani I, et al. Identification of Streptococcus suis Meningitis through Population-Based Surveillance, Togo, 2010-2014. Emerging infectious diseases. 2016;22(7):1262-4. Epub 2016/06/18. doi: 10.3201/eid2207.151511. PubMed PMID: 27314251; PubMed Central PMCID: PMCPMC4918179.

10. Toprak D, Soysal A, Torunoglu MA, Turgut M, Turkoglu S, Pimenta FC, et al. PCR-based national bacterial meningitis surveillance in Turkey: years 2006 to 2009. The Pediatric infectious disease journal. 2014;33(10):1087-9. Epub 2014/11/02. doi: 10.1097/inf.0000000000000378. PubMed PMID: 25361189.

11. Ceyhan M, Ozsurekci Y, Gurler N, Karadag Oncel E, Camcioglu Y, Salman N, et al. Bacterial agents causing meningitis during 2013-2014 in Turkey: A multi-center hospital-based prospective surveillance study. Human vaccines & immunotherapeutics. 2016;12(11):2940-5. Epub 2016/07/28. doi: 10.1080/21645515.2016.1209278. PubMed PMID: 27454468; PubMed Central PMCID: PMCPMC5137527.
