# Supplementary material for: Global etiology of bacterial meningitis: A systematic review and meta-analysis
Source: PLoS One. 2018 Jun 11;13(6):e0198772. doi: 10.1371/journal.pone.0198772 (PMC5995389; doi:10.1371/journal.pone.0198772)
Supplement: S5 Table — n, number of studies; NA, not applicable; NR, not reported. (DOCX) [file pone.0198772.s007.docx]

**S5 Table. Overview of the number of studies on frequency of pathogens that caused bacterial meningitis in all ages in Northern America (USA, Canada, Greenland).**

|  | ***E. coli***  **(n=0)** | ***H. influenzae***  **(n=2)** | ***L. monocytogenes***  **(n=1)** | ***N. meningitides***  **(n=2)** | ***S. aureus***  **(n=0)** | ***S. agalactiae* group B**  **(n=1)** | ***S. pneumoniae***  **(n=2)** |
| --- | --- | --- | --- | --- | --- | --- | --- |
| **Frequency of pathogen, weighted mean, % (95% CI)** | NA | 6.7  (6.5–6.9) | NA | 25.2  (24.8–25.6) | NA | NA | 43.1  (42.6–43.5) |
| **I^2^ (p-value)** | NA | NR | NA | NR | NA | NA | NR |

n, number of studies; NA, not applicable; NR, not reported.
