# Supplementary material for: Global etiology of bacterial meningitis: A systematic review and meta-analysis
Source: PLoS One. 2018 Jun 11;13(6):e0198772. doi: 10.1371/journal.pone.0198772 (PMC5995389; doi:10.1371/journal.pone.0198772)
Supplement: S6 Table — n, number of studies; NA, not applicable. (DOCX) [file pone.0198772.s008.docx]

**S6 Table. Overview of the number of studies on frequency of *S. agalactiae* group B that caused bacterial meningitis in neonates (aged <1 month), stratified by region.**

|  | **Africa**  (n=2) | **Eastern Mediterranean**  (n=2) | **Europe**  (n=1) | **South East Asia**  (n=2) | **The Americas**  (n=0) | **Western Pacific**  (n=1) |
| --- | --- | --- | --- | --- | --- | --- |
| **Frequency of pathogen, mean or weighted mean, % (95% CI)** | 8.7  (0.6–22.0) | 4.9  (1.7–9.3) | 58.2  (50.8–65.2) | 8.5  (0.5–21.7) | NA | 37.2  (29.8–45.2) |
| **I^2^ (p-value)** | NA | NA | NA | NA | NA | NA |

n, number of studies; NA, not applicable.
